# A caveolin-dependent and PI3K/AKT-independent role of PTEN in β-catenin transcriptional activity

**DOI:** 10.1038/ncomms9093

**Published:** 2015-08-26

**Authors:** Alejandro Conde-Perez, Gwendoline Gros, Christine Longvert, Malin Pedersen, Valérie Petit, Zackie Aktary, Amaya Viros, Franck Gesbert, Véronique Delmas, Florian Rambow, Boris C. Bastian, Andrew D. Campbell, Sophie Colombo, Isabel Puig, Alfonso Bellacosa, Owen Sansom, Richard Marais, Leon C. L. T. Van Kempen, Lionel Larue

**Affiliations:** 1 Normal and Pathological Development of Melanocytes, Institut Curie, Orsay 91405, France; 2 CNRS, UMR3347 Bat. 110, Orsay Cedex 91405, France; 3 INSERM U1021, Orsay Cedex 91405, France; 4 Equipe labellisée-Ligue Nationale contre le Cancer, Orsay Cedex 91405, France; 5 Targeted Therapy Team, The Institute of Cancer Research, 237 Fulham Road, London SW3 6JB, UK; 6 Molecular Oncology Group, Cancer Research UK Manchester Institute, The University of Manchester, Wilmslow Road, Manchester M20 4BX, UK; 7 Departments of Dermatology and Pathology and UCSF Helen Diller Family Comprehensive Cancer Center, University of California San Francisco, San Francisco, California 94143, USA; 8 The Beatson Institute for Cancer Research, Glasgow G61 1BD, UK; 9 Fox Chase Cancer Center, Philadelphia, Pennsylvania 19111, USA; 10 Department of Pathology, Radboud University Nijmegen Medical Centre, Nijmegen 6500 HB, The Netherlands; 11 Jewish General Hospital, Lady Davis Institute for Medical Research, Montreal, Quebec QC H3T 1E2, Canada; 12 Department of Pathology, McGill University, Montreal, Quebec QC H3T 1E2, Canada

## Abstract

Loss of the tumour suppressor PTEN is frequent in human melanoma, results in MAPK activation, suppresses senescence and mediates metastatic behaviour. How PTEN loss mediates these effects is unknown. Here we show that loss of PTEN in epithelial and melanocytic cell lines induces the nuclear localization and transcriptional activation of β-catenin independent of the PI3K–AKT–GSK3β axis. The absence of PTEN leads to caveolin-1 (CAV1)-dependent β-catenin transcriptional modulation *in vitro*, cooperates with NRAS^Q61K^ to initiate melanomagenesis *in vivo* and induces efficient metastasis formation associated with E-cadherin internalization. The CAV1-β–catenin axis is mediated by a feedback loop in which β-catenin represses transcription of *miR-199a-5p* and *miR-203*, which suppress the levels of *CAV1* mRNA in melanoma cells. These data reveal a mechanism by which loss of PTEN increases CAV1-mediated dissociation of β-catenin from membranous E-cadherin, which may promote senescence bypass and metastasis.

## Abstract (web summary)

The mechanism by which PTEN mutation is melanomagenic is complicated by different PTEN functions in different cellular locations. Here, the authors identify an alternative to membrane PI3K–AKT signalling, a caveolin-1-dependent PTEN pathway that induces nuclear localization and activation of β-catenin.

## Graphical abstract

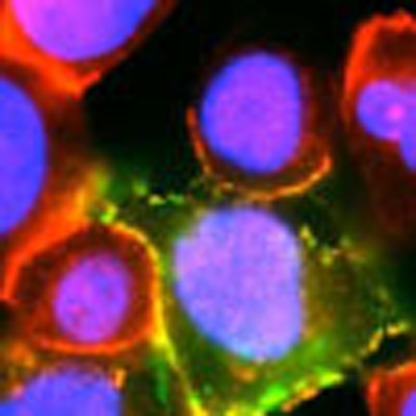

Melanomagenesis is a multistep process including initiation and progression. Mutant BRAF- and NRAS-driven mitogen-activated protein kinase (MAPK) signalling promotes proliferation of melanocytes, but this is effectively blunted by the induction of cellular growth arrest known as oncogene-induced senescence (OIS)^1^,^2^,^3^. The cell cycle inhibitor p16^INK4A^ is critical for this process and its expression is induced by the histone demethylase JMJD3 (ref. 4). OIS is bypassed in melanoma via loss of the *p16*^*INK4A*^ gene or suppression of its transcription by nuclear β-catenin^2^,^3^,^5^,^6^. Hemizygous phosphatase and tensin homologue (*PTEN*) loss is frequent in various cancers. Mutational inactivation and/or deletion of *PTEN* is found in about 20% of uncultured primary and metastatic melanomas^7^,^8^,^9^,^10^ and in 30%–40% of melanoma cell lines^9^. In melanoma tissue, loss of PTEN protein expression has been observed in ∼15% of the cases^7^,^11^, but hemizygous gene loss has been observed to be occurring more frequently, that is, 34% (ref. 7). *PTEN* loss in nevi is rare, that is, 2 out of 39 (ref. 12), suggesting that *PTEN* aberrations in melanocytes are unlikely to contribute to their uncontrolled proliferation. In *Dct::Cre* mice, the inactivation of both *PTEN* alleles does not lead to a difference in the number of nevi^13^. Altogether, it is unlikely that altered PTEN expression directly stimulates abnormal proliferation of melanocytes, but the exact contribution of PTEN to melanoma development and progression remains poorly understood.

Epigenetic inactivation or loss of *PTEN* may occur at different stages of melanomagenesis, but remains controversial for its role in senescence. On one hand, the acute loss of PTEN and APC/FZR1 induces senescence in mouse primary fibroblasts^14^. However, the inactivation of *PTEN* failed to induce a robust growth arrest in human IMR90 fibroblasts^15^. Moreover, in human BRAF^V600E^-mutated melanocytes, reducing PTEN expression was sufficient to bypass senescence^16^. In mice, the induction of a *BRAF* mutation after birth induces nevi formation and melanomas arise harbouring deletion of *p16*^*INK4A*^ or *PTEN*^1^,^17^. These results suggest that the lack of PTEN or p16INK4A contributes to the bypass of senescence *in vivo*. PTEN has different functions depending on its subcellular localization^18^. At the membrane it can dephosphorylate phosphatidylinositol (3,4,5)-triphosphate, thereby regulating AKT phosphorylation and activity. Among other functions, cytoplasmic PTEN has been shown to interact with caveolin-1 (CAV1), a major endocytic protein in mammals^19^. Such PTEN–CAV1 interaction could implicate this phosphastase in cell signalling other than the canonical PI3K–AKT–GSK3β axis.

In this study, we uncovered a signalling mechanism by which PTEN affects nuclear localization and transcriptional activity of β-catenin through a reciprocal interplay with CAV1. We discovered that the lack of PTEN, through CAV1, induces β-catenin transactivation, leading to the repression of p16^INK4A^. The co-occurrence of *NRAS*^*G183T*^ mutation and PTEN loss was detected in a fraction of human melanoma biopsies, suggesting a non-epistatic mechanism. Indeed, in a mouse melanoma model, hemizygous *PTEN* loss synergized with *NRAS* mutation and led to bypass of senescence. Thus, we have identified a novel CAV1-dependent pathway by which PTEN affects β-catenin activity and mediates melanomagenesis.

## Results

### PTEN affects β-catenin nuclear localization

**Figure 1:**
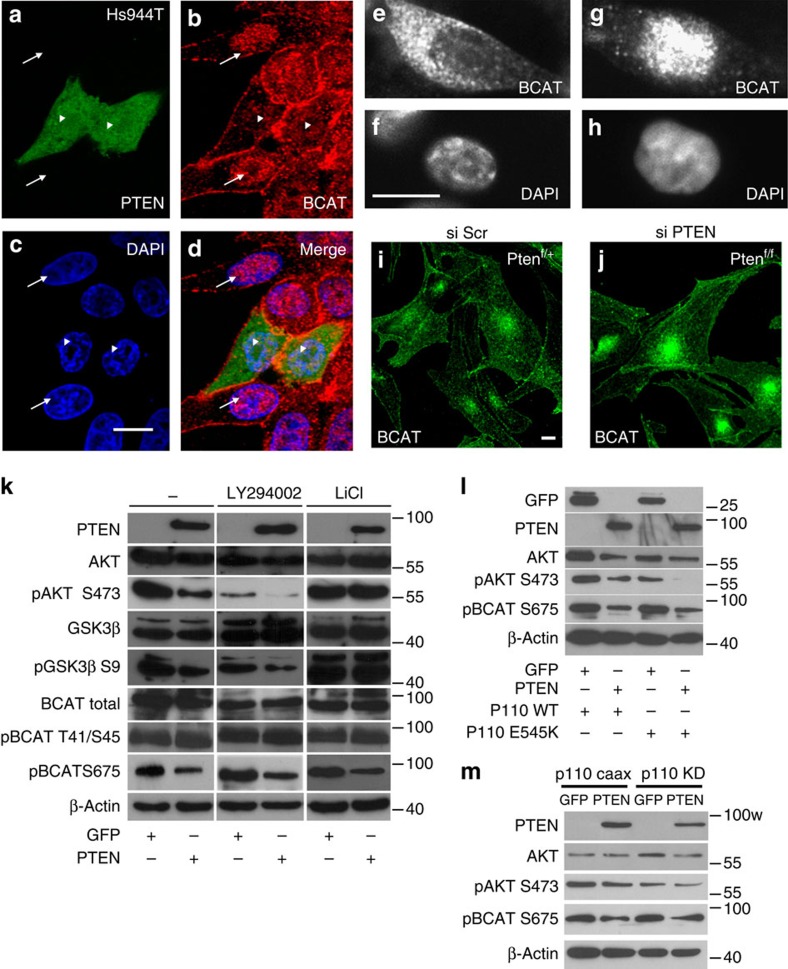
PTEN affects β-catenin nuclear localization. (**a**) Confocal microscopy revealed cells (labelled with arrows) with a heavily laden β-catenin (bcat) nuclear staining (**b**), in contrast to nearby PTEN-GFP-positive cells, where β-catenin staining could seldom be observed within the nucleus, arrowheads. Cells were counterstained with 4,6-diamidino-2-phenylindole (DAPI) (**c**). Merged is shown (**d**). Scale bar, 10 μm. Immunofluorescence experiments for the Hs944T cells were performed three times with similar results. (**e**–**h**) Human melanoma Lyse cells mutated for NRAS (Q61K), which produce PTEN, were transfected with siScr (**e**,**f**) and siPTEN (**g**,**h**). Cells were labelled for β-catenin (**e**,**g**) and counter stained with DAPI (**f**,**h**). Scale bar, 10 μm. Immunofluorescence experiments for the Lyse cells were performed two times with similar results. (**i**,**j**) Confocal microscopy showing the localization of β-catenin in *Tyr::Cre/°;PTEN*^*f/+*^*=PTEN*^*f/+*^ (**i**) and *Tyr::Cre/°;PTEN*^*f/f*^*=PTEN*^*f/f*^ (**j**) melanocytes. Note the increase of nuclear β-catenin staining in *PTEN*^*f/f*^ cells. Scale bar, 10 μm. Immunofluorescence experiments for the murine *PTEN*^*f/+*^ and *PTEN*^*f/f*^ cells were performed four times with similar results. (**k**) Immunoblot analysis of PTEN, AKT (total and phosphorylated form Ser473), GSK3β (total and phosphorylated form Ser9), β-catenin (total, phosphorylated form Thr41/Ser45 and Ser675) and β-actin proteins in Hs944T transfected with either expression vector encoding GFP (CMV::GFP) or PTEN (CMV::PTEN-GFP). Cells expressing either exogenous GFP or PTEN treated with LY294002 or LiCl for 1 h. It is noteworthy that a higher concentration of GSK3β antibody reveals a second upper band. Western blot analyses were performed two times for all antibodies with similar outputs. (**l**) Immunoblot blot analysis for GFP, PTEN, AKT (total and pSer473), β-catenin pSer675 and β-actin of Hs944T lysates co-transfected with either empty vector GFP or PTEN and WT or constitutively active p110 E545K mutant. Cells were starved for 2 h with 0.1% serum before lysis. Western blot analyses were performed two times for all antibodies with similar outputs. (**m**) Immunoblot blot analysis for PTEN, AKT (total and pSer473), β-catenin pSer675 and β-actin of Hs944T lysates co-transfected with a constitutive active form of p110 (p110 CAAX) or a kinase-dead form (p110 KD) in the presence of GFP or PTEN. Western blot analyses were performed three to six times, depending on the antibody with similar outputs.

To explore the possibility that PTEN induces re-localization of β-catenin from the plasma membrane to the nucleus, we transiently re-expressed PTEN in human PTEN^null^ human cells (Hs944T) (Fig. 1a–d). In non-transfected cells, β-catenin was localized in the nucleus. On PTEN expression, the level of β-catenin in the nucleus was significantly diminished, ∼60% of green fluorescent protein (GFP)-transfected cells compared with 20% for PTEN (Supplementary Fig. 1a). In addition, we performed subcellular fractionation experiments on GFP- and PTEN-transfected Hs944T cells. Consistent with immunofluorescence assays, the levels of nuclear β-catenin were lower in PTEN-Hs944T cells compared with GFP-Hs944T cells (Supplementary Fig. 1b). Conversely, small interfering RNA (siRNA)-mediated PTEN knockdown in PTEN^wt^ human Lyse melanoma cells, as shown by western blot analysis (Supplementary Fig. 1c), resulted in increased translocation of β-catenin into the nucleus from 40% compared with 2% in control cells (Fig. 1e–h and Supplementary Fig. 1d). These results mimic the observation from murine melanocytes lacking PTEN, which exhibit strong nuclear β-catenin localization (Fig. 1i,j and Supplementary Fig. 1e). One possible explanation for the relationship between PTEN loss and nuclear β-catenin localization is that the latter is a consequence of activation of the PI3K–AKT axis and inhibition of GSK3β. Thus, we evaluated the PI3K–AKT–GSK3β axis in relationship to the level of pThr41-Ser45 β-catenin to explain its nuclear localization (Fig. 1k). Re-expression of PTEN affected the activity of downstream effectors of phosphoinositide 3-kinase (PI3K), as indicated by the reduction of pAKT (Ser473) and pGSK3β (Ser9), but did not affect the level of total AKT and GSK3β. Even though the level of pThr41/Ser45 β-catenin was similar, on PTEN re-expression the total amount of β-catenin was slightly reduced and the quantity of transcriptionally active form of pβ-catenin (Ser675) was decreased, explaining the lower β-catenin nuclear staining. This indicated that the observed strong changes in β-catenin localization could not be explained by minor molecular changes, if any, in the destruction complex that targets β-catenin for degradation. These results were confirmed on pharmacological inhibition of PI3K or GSK3β, using LY294002 and LiCl treatment, respectively, in cells that were transfected or not with PTEN. The decrease of pSer675 β-catenin by PTEN transfection was observed even in the presence of these compounds. LY294002 treatment efficacy was demonstrated by a decrease in pAKT (Ser473) and pGSK3β (Ser9) levels. Positive controls for LiCl treatment included lack of modification of the level of pAKT Ser473 in the presence of LiCl (certainly owing to the resultant of two effects, the dephosphorylation of AKT by PTEN and the induction of phosphorylation of AKT by LiCl^20^) and induction of pGSK3β (Ser9) irrespective of the presence of PTEN. Moreover, we observed a consistent reduction of pβ-catenin Ser675 levels after concomitant re-expression of PTEN and wild-type (WT) p110 or constitutive active p110 mutant (E545K) (Fig. 1l and Supplementary Fig. 1f). Furthermore, similar results were obtained using a different constitutive active form of p110 (p110 CAAX) and a kinase-dead form of p110 (p110 KD) (Fig. 1m). Lastly, we quantified the amount of cells with positive β-catenin nuclear staining after transfection with GFP, WT PTEN, catalytically inert C124S, lipid (G129E) and protein phosphatase (Y138L) mutants, respectively (Supplementary Fig. 1g). Altogether, these results suggest that in the absence of PTEN, pathways other than PI3K–AKT and GSK3β are involved in the nuclear localization of β-catenin and the accumulation of active pSer675 β-catenin.

### PTEN inhibits the CAV1/β-catenin immunocomplex

**Figure 2:**
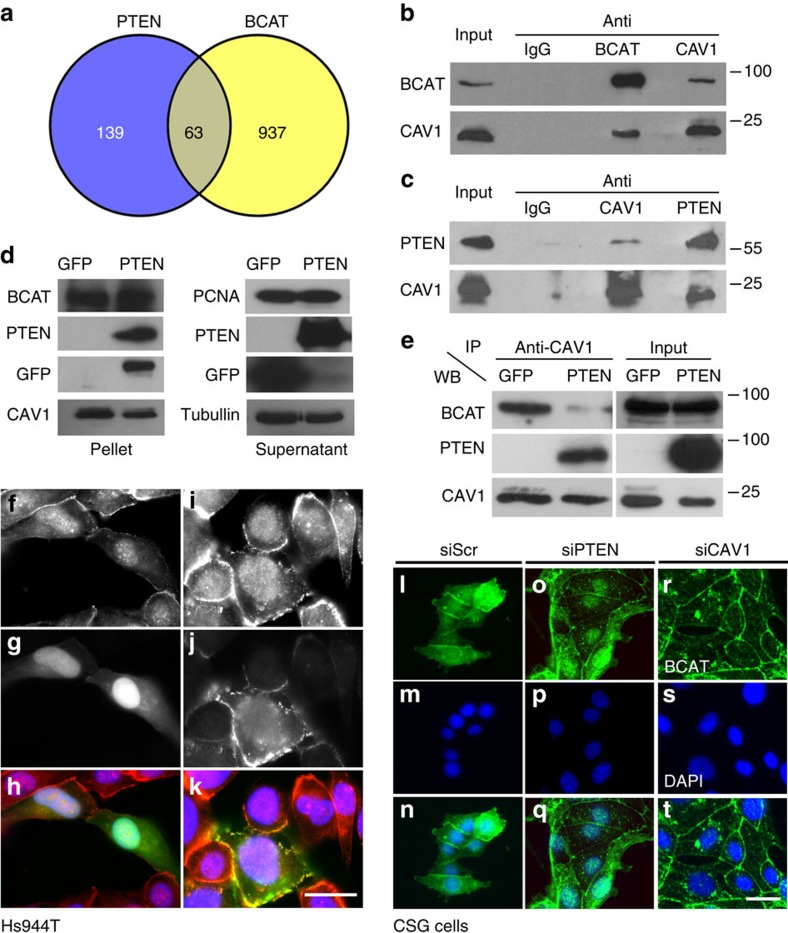
PTEN inhibits the CAV1/β-catenin immunocomplex. (**a**) Interactome of PTEN and β-catenin (BCAT) as determined by the *in silico* Ingenuity Pathway Analysis (IPA). The Venn diagram reveals 63 common members. Interaction of CAV1 with β-catenin (BCAT; **b**) and PTEN (**c**) in Rosi human melanoma cells. Extracts from ∼1.5 × 10^7^ cells (corresponding to about 1.5 mg) were immunoprecipitated with control IgG, anti-BCAT, anti-CAV1 or anti-PTEN antibodies. Immune complexes were resolved by SDS–PAGE and blotted with antibodies to BCAT, CAV1 and PTEN. Total protein input corresponds to 2% of the total protein used for immunoprecipitation. One-tenth of the IP sample was loaded to detect BCAT following IP with BCAT antibodies (and similarly for CAV1 and PTEN). This allowed getting a reasonable intensity for the corresponding signals. For the other lanes, the entire IP samples were loaded. (**d**) GST pulldown using β-catenin–GST fusion Sepharose beads and whole-cell protein lysates (500 μg) from Hs944T cell transfection with GFP or PTEN. Pellet and supernatant fractions were immunoblotted for various antibodies. (**e**) CAV1-BCAT immunocomplex in GFP transiently transfected Hs944T cells. When transfected with PTEN, the proportion of β-catenin in the immunocomplex is dramatically reduced. Total protein input is shown. For **b**–**e**, experiments were performed three times. (**f**–**k**) Immunofluorescence of transiently transfected Hs944T human melanoma cell line with *CMV::GFP* and *CMV::CAV1-RFP* (CAV1) expression vector. β-Catenin staining (**f**,**i**), GFP (**g**), CAV1 (**j**) and merged (**h**,**k**). Scale bar, 25 μm. Immunofluorescence experiments for Hs944T cells were performed three times with similar results. (**l**–**t**) Immunofluorescence of mouse carcinoma submandibular gland (CSG) cells transfected with siRNA directed against either negative control (siScr), PTEN (siPTEN) and CAV1 (siCAV1) stained for BCAT (**l**,**o**,**r**), respectively. Cells were counterstained with 4,6-diamidino-2-phenylindole (DAPI) (**m**,**p**,**s**). Merge (**n**,**q**,**t**). Scale bar, 25 μm for all panels. Immunofluorescence experiments for the CSG cells were performed two times with similar results.

According to ingenuity pathway analysis, PTEN and β-catenin share 63 interactors (Fig. 2a), including CAV1, AKT, platelet derived growth factor receptor (PDGFR), early growth response 1 (EGR1), v-erb-b2 erythroblastic leukemia viral oncogene homolog 2 (ERBB2), histone deacetylase 3 (HDAC3), enhancer of zeste homolog 2 (EZH2) and different FOXO proteins (Supplementary Data 1–3). Among them, CAV1 is found in the sub-membrane area and in the cytoplasm and it was already suggested that CAV1 might be a positive regulator of β-catenin in human gastric cancer cells^21^. CAV1-scaffolding domain interacts with either PTEN or β-catenin^19^,^22^,^23^,^24^. Thus, we hypothesized that PTEN and β-catenin could compete for CAV1, subsequently affecting different signalling outcomes. We first verified that CAV1 and β-catenin are able to immuno-complex in a reciprocal manner in Rosi human melanoma and HCT116 human carcinoma cell lines (Fig. 2b and Supplementary Fig. 2a). Similarly, we confirmed that endogenous CAV1/PTEN can immune-complex in a reciprocal fashion in Rosi and HCT116 cells (Fig. 2c and Supplementary Fig. 2a). In addition, we validated this CAV1/PTEN interaction in WM852 and WM793 human melanoma cell lines after immune-precipitation with CAV1 (Supplementary Fig. 2b).

Furthermore, using glutathione *S*-transferase-β (GST-β)–catenin fusion protein, we precipitated CAV1 on Hs944T cells transfected with GFP and, to a lesser extent, in cells expressing exogenous PTEN (Fig. 2d). Expanding on the GST pull-down assays, co-immunoprecipitation experiments in Hs944T cells expressing exogenous PTEN reveal that re-expression of PTEN significantly abrogates β-catenin/CAV1 interactions (Fig. 2e). In addition, after transfection of Hs944T cells using several PTEN-GFP constructs (WT, C124S, Y138L and G129E) we observed that the PTEN/CAV1 interaction is unaffected by the C124S mutant compared with WT, but highly disturbed by the Y138L and G129E mutants as revealed after immunoprecipitation experiments using CAV1 antibody (Supplementary Fig. 2c,d). As previously stated (Supplementary Fig. 1a), β-catenin is observed ∼60% of the time within the nucleus of Hs944T cells. On overexpression of GFP or CAV1 in Hs944T cells, immunofluorescence analysis revealed nuclear β-catenin staining, similar to controls (Fig. 2f–k and Supplementary Fig. 1a). Treatment of the Hs944T cells with LY294002 did not affect the level of β-catenin in the cytoplasm or in the nucleus when overexpressing CAV1, confirming that PI3K pathway has limited function in the nuclear translocation of β-catenin under these conditions (Supplementary Fig. 2e–j). In murine epithelial CSG cells, expressing PTEN, CAV1 and β-catenin, the latter is found at cell–cell contacts, in the cytoplasm and in the nucleus, once the cells form small islets. In these conditions, the reduction of PTEN leads to a nuclear β-catenin (7.5% to 46%) and the reduction of CAV1 leads to a recruitment of β-catenin at the cell–cell contacts, and a reduction of nuclear β-catenin, from 7.5% to 2% (Fig. 2l–t and Supplementary Fig. 2k). Similar experiments were performed with murine pancreatic epithelial cells expressing PTEN (KPC1) or not (KCPTEN2). The reduction of PTEN and CAV1 in KPC1 cells was demonstrated by western blot analysis (Supplementary Fig. 2a). Concurrently, decreased amount of PTEN led to increased nuclear β-catenin of cells, from 20% to 50%, whereas the diminution of CAV1 resulted in an accumulation at the cell–cell contacts of β-catenin and nuclear exclusion, from 20% to 6% of cells (Supplementary Fig. 3a,b). As expected, β-catenin is mainly nuclear in KCPTEN2 cells (Supplementary Fig. 3c,d). Re-expression of PTEN in KCPTEN2 cells led to an accumulation of β-catenin at cell–cell contacts and nuclear exclusion from 18% of PTEN-expressing cells compared with 91% in controls (Supplementary Fig. 3d). The overexpression of CAV1 in KCPTEN2 cells did not affect the localization of β-catenin. The absence of response of β-catenin is certainly due to the already high level of CAV1 in KCPTEN2 cells. Furthermore, CAV1 overexpression in PTEN-re-expressing cells failed to rescue β-catenin nuclear exclusion, consistently with immunoprecipitation experiments where PTEN significantly abrogates CAV1/β-catenin complex (Fig. 2e and Supplementary Fig. 1a). Thus, it appears that PTEN affects β-catenin-CAV1 complex and decreases the level of nuclear β-catenin.

### CAV1 regulates the transcriptional activity of β-catenin

**Figure 3:**
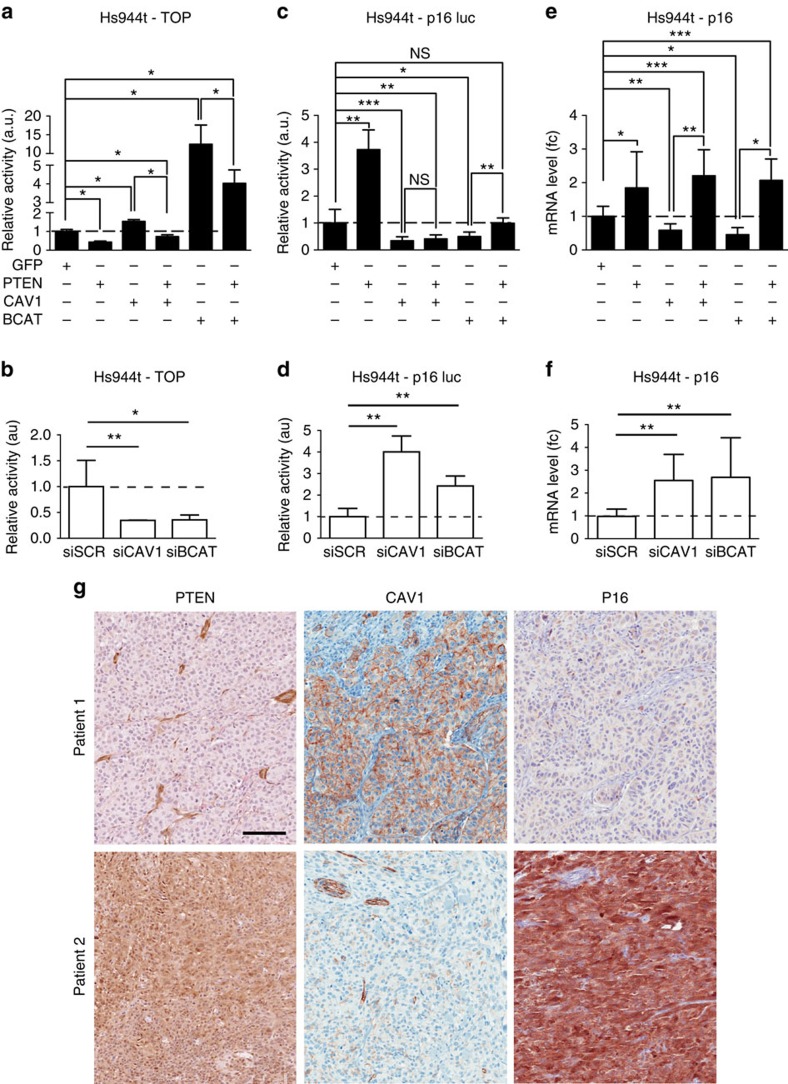
CAV1 regulates the transcriptional activity of β-catenin. (**a**) TOP-FLASH activity in Hs944T cells in the presence of GFP, PTEN and/or CAV1 β-catenin (BCAT). (**b**) Similarly, TOP-FLASH activity was measured in the same cells post transfection of siRNA directed against negative control (siSCR), CAV1 (siCAV1), β-catenin (siBCAT). (**c**) Activity of p16INK4A::luciferase reporter was evaluated post transfection in Hs944T human melanoma cells with GFP, PTEN and/or CAV1 BCAT, and (**d**) with siSCR, siCAV1 and siBCAT. (**a**–**d**) All *p16INK4A::luciferase* and TOP-FLASH reporter assays were evaluated in the presence of an internal control (*Renilla* luciferase). (**e**,**f**) *p16* mRNA level as measured by quantitative reverse transcriptase–PCR (fold change), following overexpression of GFP, PTEN and/or CAV1 BCAT, or knockdown of CAV1 and BCAT, with appropriate controls. (**g**) Eighteen human melanoma tumours were stained for CAV1, PTEN and p16. PTEN and p16 were absent and CAV1 was present for patient 1. Opposite observation was performed for patient 2. Stromal and endothelial cells were used as positive control for PTEN and CAV1, respectively. Scale bar, 100 μm for all panels. Error bars represent s.d. *P-value <0.05, **P-value <0.01 and ***P-value <0.001. Statistical significance was determined by Mann–Whitney test. Each experiment was performed in eight and three biological triplicates for **a**–**d**, **e** and **f** respectively.

We first assessed whether PTEN and CAV1 expression affects the transcriptional activity of β-catenin, using the TOP flash reporter assay in Hs944T cells. TOP flash activity is significantly reduced in the presence of PTEN and conversely induced with CAV1 (or β-catenin as positive control) (Fig. 3a). Co-expression of either PTEN and CAV1 or PTEN and BCAT resulted in a significant decrease in TOP flash activity, restoring basal-like levels on PTEN and CAV1 expression and, to a lesser extent, on PTEN and beta-catenin (BCAT) expression. Consistently, TOP flash activity decreased on CAV1 or β-catenin downregulation using appropriate siRNAs (Fig. 3b). In addition, we observed a similar effect on TOP flash reporter after modulating the levels of CAV1 and BCAT in a BRAF (BRAF^V600E^) PTEN-null cell line (Supplementary Fig. 4a,b). The transcriptional activity of *MITF-M*, the master gene of the melanocyte lineage and a known β-catenin transcriptional target, was induced after overexpression of CAV1 and BCAT (Supplementary Fig. 4c). Owing to the fact that β-catenin can also act as a co-transcriptional repressor, we performed similar experiments for p16^INK4A^. PTEN overexpression significantly induced p16^INK4A^ luciferase reporter activity (Fig. 3c). Overexpression of CAV1 or BCAT significantly reduced p16^INK4A^ luciferase reporter activity, whereas inverse knockdown significantly increased reporter activity (Fig. 3c,d). PTEN overexpression completely rescued the inhibitory effect of BCAT on p16^INK4A^ luciferase reporter (Fig. 3c). However, under these conditions, PTEN overexpression failed to rescue the inhibitory effect of CAV1 on p16^INK4A^ luciferase reporter. As expected, PTEN overexpression increased p16 messenger RNA transcript, whereas CAV1 and BCAT reduced the levels (Fig. 3e). We also observed an increase in p16 mRNA on co-transfection of PTEN and CAV1 or PTEN and BCAT to similar levels as PTEN alone. In congruency with prior results, knockdown of CAV1 or BCAT resulted in an increase of p16 mRNA levels (Fig. 3f). We then wondered whether the levels of CAV1 could also modulate *MYC*, another β-catenin target gene. Indeed, MYC levels were directly affected by altering CAV1 (Supplementary Fig. 4d,e). Finally, in agreement with our *in vitro* data, histomolecular analysis of human melanoma biopsies revealed the existence of PTEN-negative, CAV1-positive and P16-negative tumour (Fig. 3g). In conclusion, CAV1 acts on the transcriptional activity of exogenous (TOP) and endogenous (MITF, MYC and p16^INK4A^) β-catenin targets.

### NRAS^Q61K^ and PTEN loss cooperate during melanoma initiation

**Figure 4:**
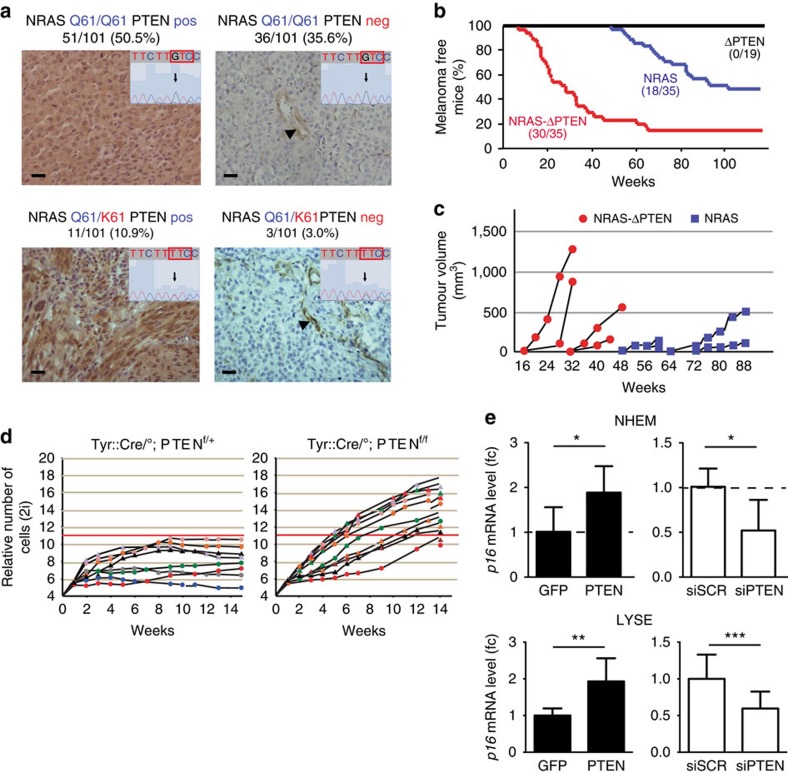
NRAS^Q61K^ and PTEN loss cooperate during melanoma initiation. (**a**) Human melanoma library containing 101 samples was subjected to immunohistochemical analysis for PTEN expression alongside NRAS mutational status. For each case type: the minimized window in the foreground represents the DNA sequence of NRAS at codon 61, while the background picture is the corresponding PTEN immunostaining. Arrowheads indicate blood vessels, internal positive control for PTEN expression. Vertical arrows indicate the location of the G to T mutation. Scale bar, 100 μm. (**b**) Kaplan–Meier (KM) melanoma-free mice analysis of ΔPTEN (*n*=19), NRAS (*n*=35) and NRAS-ΔPTEN mice (*n*=35). The KM curves between NRAS and NRAS-ΔPTEN are significantly different (*P*<10^−5^) using the Mantel–Cox test. NRAS=Tyr::NRAS^Q61K^/°, ΔPTEN=Tyr::Cre/°; PTEN^f/+^ and NRAS-ΔPTEN=Tyr::NRAS^Q61K^/°; Tyr::Cre/°; PTEN^f/+^. The mean number of melanomas per mouse was 1.6 and 2.1 for NRAS and NRAS-ΔPTEN mice, respectively, with a *P*-value of 0.37 (Student's *t*-test). (**c**) Tumour growths were measured and tabulated. The rate of growth of four representative tumours from NRAS and NRAS-ΔPTEN were plotted in relation to size during an 88-week interval. NRAS-ΔPTEN tumours averaged a steeper and earlier growth rate in comparison with NRAS controls. (**d**) Melanocytes were established from the skins of Tyr::Cre/°; PTEN^f/+^ and Tyr::Cre/°; PTEN^f/f^ pups. Cells were directly counted every week and the growth curve was plotted as relative number of cells in log2 form (see Delmas *et al.*^32^). Each curve corresponds to the culture established from the back skin of a single pup. Cells with biallelic disruption of the PTEN gene, *PTEN*^*f/f*^, bypassed efficiently senescence when comparing the heterozygous, PTEN^f/+^. (**e**) *p16* mRNA as measured by quantitative reverse transcriptase–PCR (fold change) transfection in normal human epithelial melanocyte (NHEM) (top panel) and Lyse human melanoma cell line (bottom panel) with GFP and PTEN expression vectors and with scramble (siScr) and PTEN (siPTEN) siRNA. Error bars represents.d. *P-value <0.05, **P-value <0.01 and ***P-value <0.001. Statistical significance was determined by Mann–Whitney test. Experiments were performed in biological triplicates.

The oncogenic form of NRAS (NRAS^Q61K/R^) and the lack of PTEN are found in ∼20% and 30% of human melanoma, respectively, and it has generally been assumed that they are mutually exclusive^10^. We found that NRAS mutation and the loss of PTEN may coexist in human melanoma. A series of 105 human melanoma samples was analysed by comparative genomic hybridization for *PTEN* loss and for the presence or the absence of point mutations affecting *NRAS*. *NRAS*^*G183T*^ mutation, resulting in an amino-acid change Q61K, was found in 16 samples (15%), of which 2 also showed homozygous *PTEN* loss (Supplementary Fig. 5a). A second independent series of 101 human melanoma samples was analysed for *NRAS*^*G183T*^ mutation and PTEN protein expression. Allele-specific PCR and DNA sequencing revealed that 14 samples harboured *NRAS*^*G183T*^ mutation. Immunohistochemistry analysis showed that 39 samples were negative for PTEN; 3 of these also contained the *NRAS*^*G183T*^ mutation (Fig. 4a). In addition, we tested the status of NRAS and PTEN in human melanoma cell lines. Lyse and Rosi cells express PTEN, whereas Hs944T and SK29 cells do not; Lyse and Hs944T cells carry *NRAS*^*G183T*^ mutation, whereas Rosi and SK29 cells are WT for *NRAS* (Supplementary Fig. 5b). Moreover, we determined the status of PTEN and p16 at the genomic, transcript and protein level for several human melanoma cell lines (Supplementary Fig. 5c–e). In conclusion, the presence of *NRAS* mutation and PTEN loss is not mutually exclusive in melanoma.

**Figure 5:**
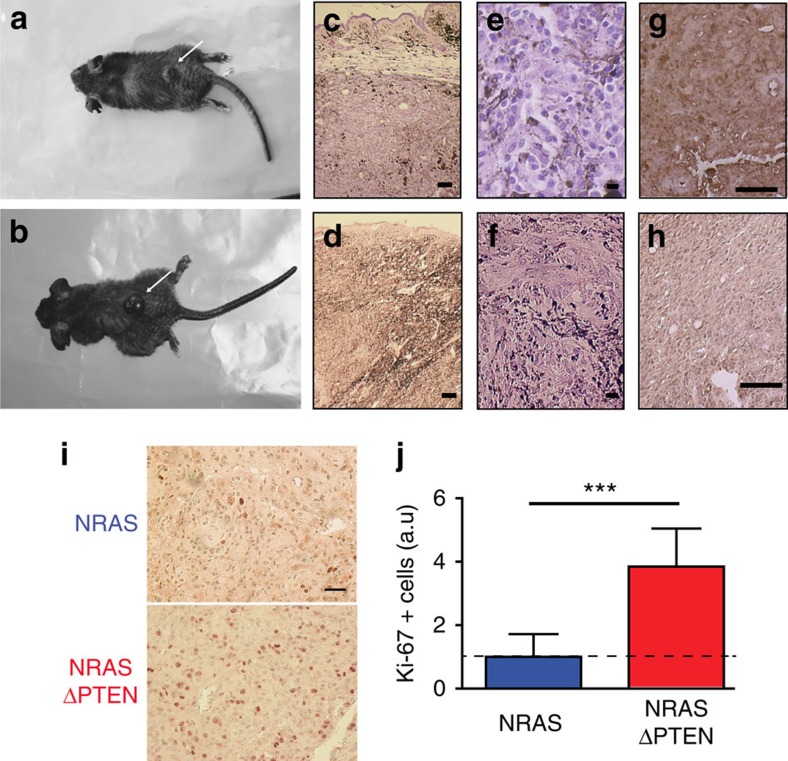
NRAS ΔPTEN melanoma characteristics. (**a**,**b**) Dorsal melanoma appearing in NRAS (**a**) and NRAS-ΔPTEN (**b**) mice (arrow). Melanoma arose from different part of the body including hairy part, pinnae and tails/paws (see Supplementary Table 1). (**c**–**f**) Haematoxylin and eosin staining of an NRAS (**c**,**e**) and NRAS-ΔPTEN (**d**,**f**) cutaneous melanoma. (**e**,**f**) Higher magnification reveals irregularly shaped pigmented cells with diverse sizes and large nuclei. (**g**,**h**) Positive immunostaining for melanoma marker S100 in NRAS and NRAS-ΔPTEN tumours. (**i**) Positive Ki-67 staining of NRAS and NRAS-ΔPTEN tumour sections. (**j**) Graphical representation of the relative number of Ki-67-positive cells in NRAS and NRAS-ΔPTEN tumour sections. The relative number of Ki-67-positive cells correspond to the ratio of the number of Ki-67+ cells from identical surface in NRAS or NRAS-ΔPTEN tumour sections versus the number of Ki-67+ cells from identical surface in NRAS tumour sections. Statistical significance was determined by Mann–Whitney test. ***P-value <0.001. Five hundred cells were assessed from four fields and four independent experiments for each genotype. Scale bars, 100 μm (**c**,**d**), 10 μm (**e**,**f**), 50 μm (**g**,**h**) and 10 μm (**i**).

A mouse melanoma model for these two mutations was generated. *Tyr::NRAS*^*Q61K*^ mice were crossed with *Tyr::Cre* mice and *PTEN*^*f/+*^ mice. We produced the following mice: *Tyr::Cre/°;PTEN*^*f/+*^ (henceforth *ΔPTEN*), *Tyr::NRAS*^*Q61K*^*/°;Tyr::Cre/°* (*NRAS*) and *Tyr::NRAS*^*Q61K*^*/°;Tyr::Cre/°;PTEN*^*f/+*^ (*NRAS-ΔPTEN*). None of the *ΔPTEN* mice developed melanoma during 2 years of follow-up observation (Fig. 4b). Half of *NRAS* mice spontaneously developed melanomas with a latency period of 71±16 weeks. *NRAS-ΔPTEN* mice spontaneously developed melanomas, with a higher penetrance (86%) and a shorter latency (27±13 weeks) than the *NRAS* mice. Most melanomas appeared in the hairy part of the skin for both genotypes (Fig. 5a,b and Supplementary Table 1). The tumours consisted of irregularly pigmented cells with diverse sizes, large nucleoli and positive S100 immunostaining (Fig. 5c–h). Similar results were obtained with another mouse melanoma model in which the NRAS mutation is NRAS^G12D^ and the activation of the mutation occurred at 10 weeks of age in melanocytes, using the CreERt2–LoxP–tamoxifen system (Supplementary Fig. 6).

**Figure 6:**
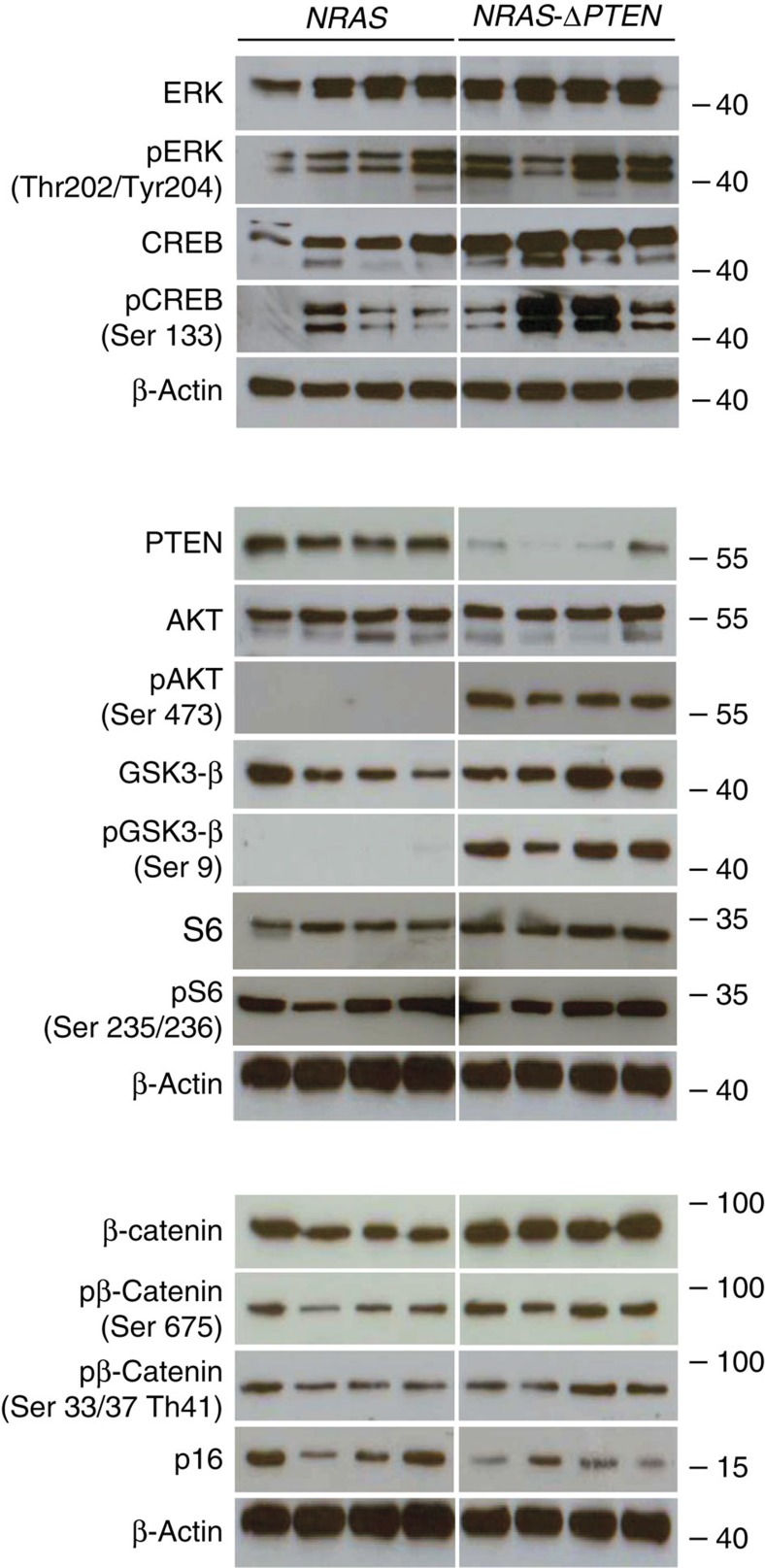
Immunoblot analysis of murine melanoma. Immunoblot analysis of the MAPK and PI3K/AKT pathways reveals differential regulation of key proteins. Immunoblot analysis of protein lysates from eight murine uncultured melanoma samples: four from NRAS and four from NRAS-ΔPTEN. Western blot analyses were performed between three and six times, depending on the antibody with similar results.

To understand the molecular mechanisms underlying the differences between *NRAS* and *NRAS-ΔPTEN* mouse melanomas and the role of PTEN in β-catenin nuclear localization, we first studied the MAPK and PI3K signalling pathways. After transformation, *NRAS-ΔPTEN* melanomas grew faster and larger than *NRAS* melanomas (Figs 4c and 5i,j). Western blot analysis revealed minimal differences in MAPK activity in *NRAS* versus *NRAS-ΔPTEN* tumours, whereas the status of the PI3K/PTEN/AKT signalling pathway was significantly different for the two genotypes (Fig. 6). PTEN was almost absent from *NRAS-*Δ*PTEN* tumour samples, suggesting that the expression of PTEN from the remaining WT allele is inhibited for unknown reasons. The amount of pAKT (Ser473) and pGSK3β (Ser9) was dramatically increased in *NRAS-ΔPTEN* compared with *NRAS*; however, most interestingly, the levels of pS6 remained unaffected. These results suggest that signalling via mammalian target of rapamycin-associated proteins was the same in *NRAS* and *NRAS-ΔPTEN* melanomas.

We proceeded to evaluate the amount of β-catenin and p16^INK4A^. In agreement with our results with melanoma cell lines, the level of pSer33/37-Thr41 β-catenin, targeted for proteasomal degradation, is largely unaltered in *NRAS-ΔPTEN* compared with *NRAS*. Conversely, the amount of total and pSer675 β-catenin, corresponding to the transcriptionally active form of β-catenin, is significantly higher in *NRAS-ΔPTEN* compared with *NRAS*. Consequently, we reproducibly observed a reduction of p16^INK4A^ protein in *NRAS-ΔPTEN* as compared with *NRAS*.

Melanoma appearance is associated with proliferation and bypass of senescence. Before transformation, melanoblasts lacking PTEN do not grow faster than WT (Supplementary Fig. 7). OIS is bypassed efficiently in the absence of PTEN. We established cultures of melanocytes from *Tyr::Cre/°; PTEN*^*f/f*^ (*Hom*), *Tyr::Cre/°; PTEN*^*f/+*^ (*Het*) and *Tyr::Cre/°; PTEN*^*+/+*^ (*WT*) mice. No obvious difference was observed between *Het* and *WT* melanocytes. The initial rates of growth of the *Het* and *Hom* melanocytes *in vitro* were indistinguishable, confirming that the absence of PTEN does not induce proliferation before transformation (Fig. 4d). Cultures of *Hom* melanocytes divided continuously and rapidly became immortalized. In contrast, *Het* melanocytes in culture stopped expanding within 4 weeks of explantation and developed a large nucleus and a flattened morphology, and accumulated melanin, hallmark features of senescence. Melanocyte cell lines could be established from 90% (9 of 10) of *Hom* newborn pup skins, but only from 28% (2 of 7) of their *Het* littermates, implying that the absence of *PTEN* from melanocytes increased the efficiency of immortalization. We confirmed that by modulating PTEN levels we affected p16^INK4A^ expression in primary normal human epithelial melanocytes as well as in transformed Lyse human melanoma cells (Figs 1i,j and 4e).

### The absence of PTEN promotes efficient metastasis formation

**Figure 7:**
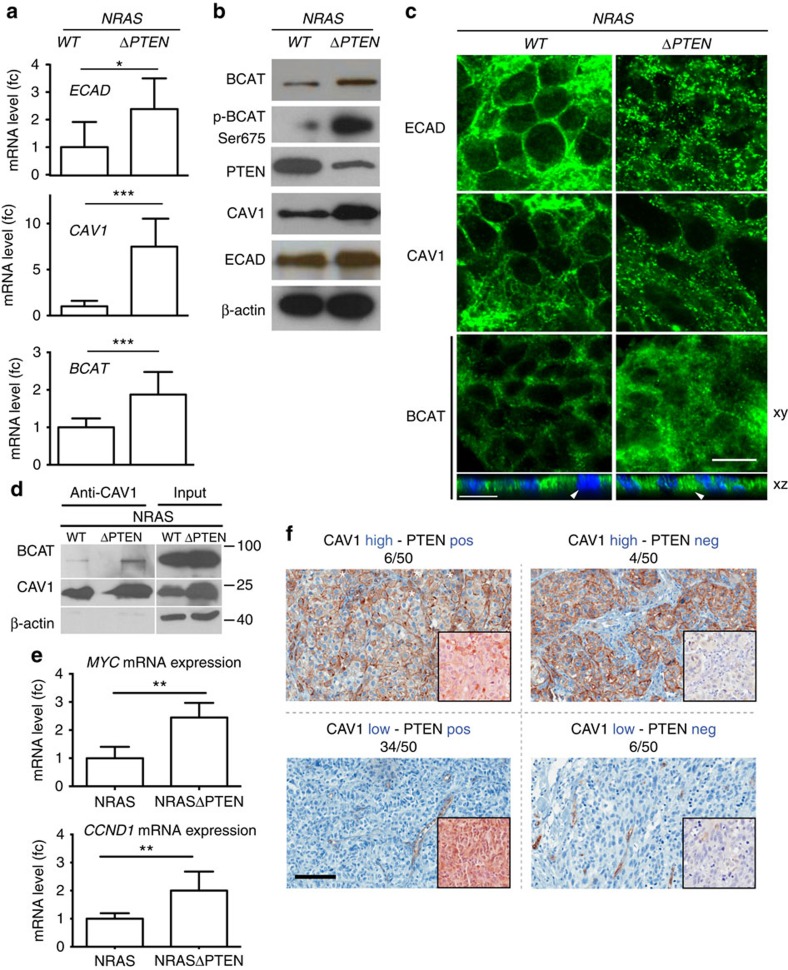
PTEN loss induces ECAD internalization and metastasis. (**a**) mRNA levels of ECAD (top), CAV1 (middle) and BCAT (bottom) in NRAS and NRAS-ΔPTEN mouse melanoma as measured by quantitative reverse transcriptase–PCR (fc, fold change). Experiments were performed three times. Error bars represent s.d. *P-value <0.05, **P-value <0.001. Statistical significance was determined by Mann–Whitney test. (**b**) Western blot analysis of uncultured whole tumour lysates from NRAS and NRAS-ΔPTEN using antibodies against BCAT (total and pSer675), PTEN, CAV1, ECAD and β-actin. Western blot analyses were performed at least two times depending on antibody. (**c**) Immunofluorescence of serial NRAS and NRAS-ΔPTEN tumour sections, stained for ECAD, CAV1 and BCAT. Scale bar, 10 μm. *xz* projection from confocal image stacks of β-catenin-stained NRAS and NRAS-ΔPTEN tumours counterstained with 4,6-diamidino-2-phenylindole (DAPI). Arrowheads indicate nucleus. Scale bar, 16 μm. Immunofluorescence experiments for the NRAS and NRAS-ΔPTEN tumour cryosections were performed two times with similar results. (**d**) Interaction of CAV1 with β-catenin (BCAT) in NRAS and NRAS-ΔPTEN tumour samples. Cell lysates containing 400 μg of total proteins were subjected to IP with an anti-CAV1 antibody. The co-IP of β-catenin with CAV1 was detected by western blotting (WB) using anti-β-catenin and anti-CAV1 antibodies. Total protein input is shown. This experiment was performed twice. (**e**) mRNA levels of downstream β-catenin target genes *c-MYC* and *CCND1* as measured from NRAS and NRAS-ΔPTEN mouse melanoma by quantitative reverse transcriptase–PCR (fc, fold change). Error bars represent s.d. **P-value <0.01. Statistical significance was determined by Mann–Whitney test. (**f**) CAV1 and PTEN were detected by immunohistochemistry on a series of 50 human melanoma sections from the second cohort. Sections of patients were either AEC stained (red) for CAV1 (background image) or PTEN (foreground image, lower-right corner) and counterstained with haematoxylin (blue). Staining for CAV1 was scored as high or low, while the amount of PTEN as positive (pos) or negative (neg). Among 50 melanoma biopsies, 34 presented a low amount of CAV1 and a positive staining for PTEN (34/50). Scale bar, 100 μm.

Autopsy of 7 *NRAS* and 17 *NRAS-ΔPTEN* mice carrying melanoma revealed the presence of lung metastasis in 1/7 and 8/17 mice, respectively (Supplementary Fig. 8). Molecular analysis of *NRAS* and *NRAS-ΔPTEN* tumours was performed to evaluate the level of β-catenin, PTEN, CAV1 and the cell–cell adhesion molecule and β-catenin interactor, E-cadherin (ECAD). ECAD can be internalized using caveolae^25^, and its levels and localization are affected by interaction with β-catenin^26^. Surprisingly, the amount of ECAD mRNA and protein was higher in the absence of PTEN (Fig. 7a,b). However, in the absence of PTEN, the amount of ECAD located at the cell–cell contact was much lower than the amount of cytoplasmic ECAD, which was dramatically increased (Fig. 7c). Similarly, mRNA and protein levels of CAV1 and β-catenin (total and pSer675 protein) were also increased in *NRAS-ΔPTEN* tumours compared with *NRAS* (Fig. 7a,b). In addition, CAV1 and β-catenin were mostly delocalized from the membrane in *NRAS-ΔPTEN* tumours (Fig. 7c). Furthermore, we confirmed that PTEN expression decreases CAV1/BCAT immuno-complex in murine tumour samples and affects the transcription of known β-catenin targets, MYC and CCDN1 (Fig. 7d,e).

Lastly, we examined the expression of CAV1 and PTEN in human melanomas. Fifty human melanoma samples were stained for PTEN and CAV1 on consecutive slides (Fig. 7d). PTEN and CAV1 were expressed in 40 and 10 melanomas, respectively. When present, CAV1 was mainly located at the membrane, but could also be found in the cytoplasm and seldom in the nucleus. Interestingly, in 34/50 cases, there is a strong tendency for low expression of CAV1 and high levels of PTEN. Altogether, these results show that melanoma samples lacking PTEN and expressing high level of CAV1 do exist in mouse and human melanomas.

### β-Catenin induces CAV1 through repression of miRs

**Figure 8:**
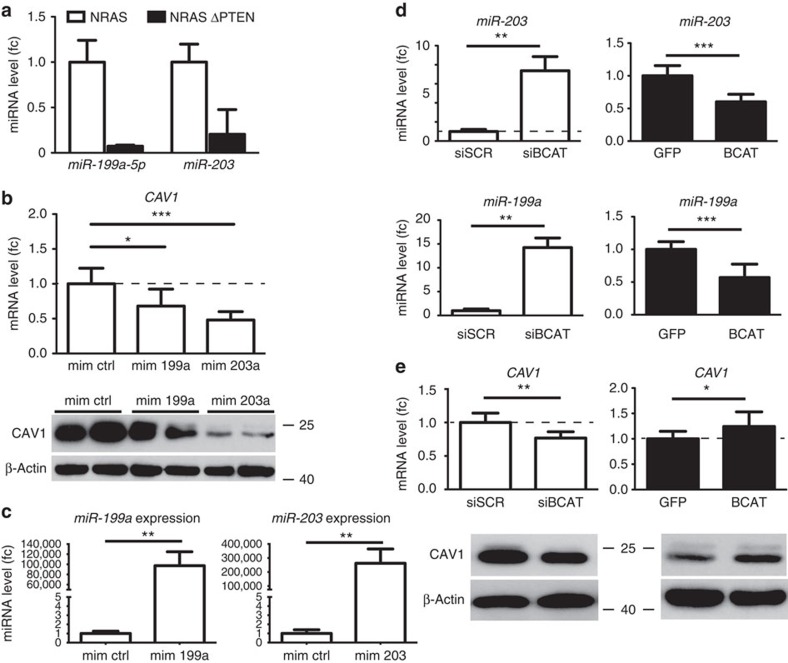
β-catenin induces CAV1 through miR-199a and miR-203. (**a**) *miR-199a-5p* and *miR-203* gene expression in NRAS and NRAS-ΔPTEN mouse tumour samples from miRNA expression arrays. (**b**) *CAV1* mRNA expression post transfection with miRNA mimics for *miR-199a* and *miR-203* in human Hs944T melanoma cells (fc, fold change). CAV1 knockdown after miRNA mimics overexpression from whole-cell protein lysates in duplicates was validated through western blotting (below). Statistical significance was determined by Mann–Whitney test. (**c**) *miR-199a-5p* and *miR-203* gene expression in Hs944T after overexpression with miR mimic 199a-5p and 203, respectively (fold change). Error bars represent s.d. **P-value <0.01. Statistical significance was determined by Mann–Whitney test. (**d**) miRNA levels as determined by quantitative reverse transcriptase–PCR (fold change) of *mir-203* and *mir-199a* in Hs944T post siRNA-mediated knockdown of β-catenin (siBCAT) or conversely overexpression of BCAT. Statistical significance was determined by Mann–Whitney test. (**e**) *CAV1* mRNA levels after either siBCAT or BCAT overexpression in Hs944T cells. Whole-cell protein lysates were analysed via western blot (see below). Error bars represent s.d. *P-value <0.05, **P-value <0.01 and ***P-value <0.001. Statistical significance was determined by Mann–Whitney test. Experiments were performed in biological triplicates.

CAV1 is regulated by miR-203 in human breast cancer cells and miR-199a-5p in lung fibroblasts^27^,^28^,^29^. A miRnome was performed on *NRAS* and *NRAS-ΔPTEN* tumours, revealed a decrease in miR-203 and miR-199a-5p in the absence of PTEN (Fig. 8a and Supplementary Data 4). We first validated that these two miRs were able to affect the amount of CAV1 mRNA in the absence of PTEN. Hs944t human melanoma cells were transfected with miR-203 and miR-199a-5p mimics, which led to the reduction of CAV1 mRNA and protein (Fig. 8b,c). Next, we wondered whether the reduction of miR expression in the absence of PTEN was related to the increased activation of β-catenin signalling. In this respect, we quantified the levels of miR-203 and miR-199a-5p after modulating β-catenin. Indeed, β-catenin represses miR-203 and miR-199a-5p transcription. (Fig. 8d). Such regulation could be direct, as chromatin immunoprecipitation (IP)experiments revealed that β-catenin binds the promoter region of miR-203. Finally, we showed that β-catenin controls the level of CAV1 mRNA and protein (Fig. 8e).

## Discussion

In this study, we demonstrate the existence of a complex signalling network involving reciprocal interactions among PTEN, CAV1 and β-catenin; regulating molecular and cellular mechanisms that play a critical role in tumour initiation and progression. PTEN is classically known to inhibit the PI3K/AKT signalling axis, but here we show that it also remarkably controls the nuclear levels and transcriptional activity of β-catenin in an alternative PI3K/AKT way. β-Catenin transcriptional activity represses p16^INK4A^ transcription, leading to bypass of senescence, and the putative tumour suppressors mir-203 and mir-199a-5p, resulting in regulation of CAV1. CAV1 interacts with either PTEN or β-catenin, modulating the localization and co-transcriptional activity of β-catenin. Importantly, PTEN loss, via CAV1 interaction, also leads to the internalization of ECAD, promoting metastasis. These events occur in epithelial (salivary and pancreatic) and non-epithelial (melanocyte) cells, and appear to be independent of the RAS-BRAF context.

Our work identifies a novel mechanism by which a subset of melanomas can escape OIS and result in aggressive tumours. This mechanism by which loss of PTEN induces bypass of senescence, allowing an earlier melanoma initiation with a higher penetrance after oncogenic NRAS^Q61K^-induced senescence, was modelled in a mouse model, relevant for human melanomagenesis. In fact, based on a small melanoma series, it was generally assumed that NRAS mutations and PTEN loss are mutually exclusive events in human melanomagenesis; however, we showed that these two events co-exist in a fraction of human melanomas (5 out of 206 melanoma from 2 independent cohorts), as it was recently showed in one case^30^. Moreover, after analysing The Cancer Genome Atlas (TCGA), we found five melanoma samples with PTEN homozygous deletion and carrying NRAS^Q61K^ mutations. Our study was limited to the PTEN loss-mediated bypass of OIS on the NRAS^Q61K^ background, but the PTEN/CAV1/β-catenin/p16^INK4A^ pathway may hold true in BRAF^V600E^ melanomas as well (Supplementary Fig. 4a–c). Moreover, in primary human fibroblasts and melanocytes, PTEN loss inhibits BRAF^V600E^ (or HRAS^V12G^)-induced senescence^15^,^16^. Consequently, the loss of PTEN results in OIS bypass associated with RAS or RAF.

Bypassing senescence is classically associated with p53/MDM and Rb/p16^INK4A^ proteins. In our models, oncogenic NRAS with or without PTEN loss did not affect neither P53, MDM2 nor MDM4 expression levels (data not shown). This supports a p53-independent model of senescence in melanoma cells in the NRAS^Q61K^ background, in which β-catenin-regulated expression of senescence-inducing p16^INK4A^ is directly affected by the absence of PTEN^6^,^31^,^32^. At this point, it has to be noted that the role of β-catenin during melanomagenesis remains controversial^33^,^34^,^35^,^36^,^37^,^38^.

The role of CAV1 in tumorigenesis is subject of debate^39^,^40^; expression is tissue specific and varies substantially depending on the stage of the disease^41^. In melanomagenesis, its function was only investigated at the level of progression, with controversial results depending likely on the molecular context^39^,^40^. We demonstrated that CAV1 immuno-complexes with β-catenin and PTEN in the melanocyte lineage. Moreover, CAV1 has been associated with accumulation of β-catenin in gastric cancer and HEK293T cells^21^,^42^. We showed that in human melanoma NRAS^Q61K^ PTEN-null cells, p16^INK4A^ is repressed through CAV1/β-catenin; this interaction is ablated on PTEN re-expression. Thus, CAV1 would serve as a promoter of tumour initiation and progression by enhancing β-catenin-related transcription.

In *NRAS-ΔPTEN* murine melanoma tumours, western blot analysis revealed that the levels of CAV1, β-catenin and ECAD were higher than in *NRAS* tumours. Moreover, it appears that ECAD is more abundant in the cytoplasm of *NRAS-ΔPTEN* melanoma cells than in *NRAS* cells, and less at the cell–cell contact (Fig. 7c). ECAD is mainly found at the cell–cell contact and can be internalized using caveolae^25^. The reduction of ECAD at the cell–cell contact is likely a feature of melanoma progression and may induce a pseudo-epithelial to mesenchymal transition.

Whereas melanoma cell lines clearly demonstrated the causal relationship between PTEN, CAV1, β-catenin and p16^INK4A^ expression to robustly bypass senescence, immunohistochemical studies of melanoma tissue revealed that this mechanism plays a role in only a fraction of cases (Fig. 7f). In fact, we observed the expected correlation trend (evaluated as *P*=0.065, using the Fisher's exact test) of CAV1^high^ PTEN^low^ or the inverse CAV1^low^ PTEN^high^ in 12 of 50 samples. Other combinations were found in the remaining samples, indicating a high level of molecular and clinico-pathological complexity that indicates that other mechanisms of OIS escape exist in human melanomagenesis.

Thus, although the validity of our model of PTEN/CAV1/β-catenin-regulated p16^INK4A^ repression is supported by our findings in cell culture, mouse models and human samples, the role of PTEN in senescence bypass is intricate and most likely context dependent. Be as it may, our studies indicate that CAV1 and CAV1-related pathways may be a potential therapeutic target for melanoma treatment. On the other hand, our findings predict that PI3K/AKT inhibitors will not block effectively the mechanism of senescence bypass caused by PTEN loss.

## Methods

### Cell culture and cell lines

Mouse primary melanocyte cell lines were established from mice 1–5 days after birth. Mice were rinsed with 70% ethanol and then in ice-cold PBS. The skin was removed and stored in PBS. Next, the skin was cut into small pieces and incubated with collagenases type 1 and 4 for 40 min at 37 °C and 5% CO_2_. Following this incubation, the dermis and epidermis were separated using forceps and the epidermis was washed in wash buffer (1x Hank's balanced salt solution, 1 mM CaCl_2_, 0.005% DNase, 20% FCS). After washing, it was centrifuged for 5 min at 1,100 r.p.m. at room temperature. The resulting cell pellet was resuspended in dissociation buffer (GIBCO) and incubated at 37 °C and 5% CO_2_ for 10 min in a petri dish. Next, the cells were put through 18- and 20-g needles and washed in a 15-ml tube with wash buffer for 10 min, allowing for the removal of grease and hairs. The supernatant was centrifuged for 5 min at 1,100 r.p.m. at room temperature (RT) and the resulting cell pellet was resuspended in PBS and counted. The cells were then centrifuged again for 5 min at 1,100 r.p.m. (room temperature) and plated in tissue-culture dishes in F12 media supplemented with 10% FCS and 200 nM 12-*O*-tetradecanoylphorbol-13-acetate.

Human melanoma cell lines were obtained from Ruth Halaban (Yale), Sylvie Robine, Florence Faure and Alain Mauviel (Institut Curie), and were previously published by the laboratory^43^,^44^,^45^,^46^,^47^,^48^. Human melanoma cell lines were grown in RPMI 1640 media (GIBCO, 21875-034), supplemented with 10% fetal bovine serum (GIBCO, 10270-106), 1% penicillin–streptomycin (GIBCO, 15140) and 1% L-glutamine (GIBCO, 25030). Normal human epidermal melanocytes were obtained from Promocell, grown and transfected according to the manufacturer's instructions (OZ Biosciences).

### Plasmid constructs

PTEN-GFP WT, PTEN-C124S-GFP, PTEN-G129E-GFP and PTEN-Y138L-GFP expression vectors were obtained^49^. A different WT pcDNA-PTEN-GFP construct was acquired from Addgene (13039)^50^. The p110 WT and E545K mutant plasmids were previously described^51^. pCDNA–β-catenin was previously described^52^. The rest of the plasmids were kindly donated by several individuals, noted in the Acknowledgement section.

### Western blotting

Whole-cell lysate was prepared from human melanoma cell lines using RIPA buffer and whole-tissue lysate was prepared from mouse melanoma tumour using SDS lysis buffer^53^. Membranes were probed with antibodies against ERK (Cell Signaling, 9102), pERK (Thr202/Thr204, Cell Signaling, 9106), CREB (Cell Signaling, 9192), pCREB (Cell Signaling, 9191S), PTEN (Cell Signaling, 9559), AKT (Cell Signaling, 2938), pAKT (Ser473, Cell Signaling 3787), GSK3-β (Santa-Cruz, sc-9166), pGSK3-β (Ser9, Cell Signaling, 9336), S6 (Cell Signaling, 2317), pS6 (Ser235/236) (Cell Signaling, 4857), β-catenin (Abcam, ab-6302), pβ-catenin (Ser675, Cell Signaling, 4176), pβ-catenin (Ser33-37/Thr41, Cell Signaling, 9561), pβ-catenin (Thr41/Ser45, Cell Signaling, 9565S), p16 (Santa-Cruz, sc-1661), CAV1 (Cell Signaling, 3238) and β-actin (Sigma, A5441). All antibodies were used at a dilution of 1/1,000, except β-actin (1/5,000). Full scans of blots accompanied by the position of the molecular weight markers are shown in Supplementary Fig. 9.

### Immunofluorescence microscopy

Primary murine melanocytes were grown to near confluence upon which point were counted, 2.5 10^5^ cells were seeded in 18mm glass cover slips and allowed 24 h to recover prior immunofluorescence analysis. Similar procedure was followed for CSG, KPC1 and KCPTEN2 cells. Human Hs944T melanoma cells were transfected with CMV::PTEN-GFP (#1031) and allowed 48 h to recover before being fixed in 4% paraformaldehyde (PFA) for 20 min at RT. Human or mouse cells were permeabilized with 0.2% v/v PBS/Triton X-100 for 5 min at RT. Then, cells were washed twice with PBS and blocked with 1% BSA (w/v) and 10% fetal bovine serum in PBS for 20 min at RT. Cells were incubated with the primary antibody anti-β-catenin (dilution 1/100) at 4 °C overnight. Alexa 555 anti-rabbit (Sigma) secondary antibody was incubated for 1 h at RT in the dark. Cells were counterstained with 0.5 μg μl^−1^ 4,6-diamidino-2-phenylindole to visualize the nucleus.

### Determination of the number of melanoblasts

Tyr::Cre/°;PTEN^f/f^, Tyr::Cre/°; PTEN^f/+^ and Tyr::Cre/°;PTEN^+/+^ mice were crossed with Dct::LacZ mice^54^ and the resulting embryos were collected at various times during pregnancy. Embryos were stained with 5-bromo-4-chloro-3-indolyl-β-D-galactoside, as previously described^55^.

### Co-immunoprecipitation

Co-IP experiments were performed as previously described^56^. After reaching 100% confluence in 150 mm tissue culture dishes, Rosi and HCT116 cells were washed two times with cold PBS on ice. After washing, cells were scraped off of the dishes in 1 ml of IP buffer (10 mM Tris (pH 8), 150 mM NaCl, 1% (v/v) Triton X-100, 60 mM Octyl β-D-glucopyranoside (Sigma, O8001), 1 × protease and phosphatase inhibitors) and were incubated on ice for 30 min. Next, the lysates were centrifuged at 13,000 r.p.m. for 10 min at 4 °C. Following centrifugation, the supernatant (extract) was removed and used for IP and the pellet discarded. Fifty microlitres of the extract was kept for a total cell lysate (Input). For each IP, 1 ml extract (corresponding to ∼1.5 × 10^7^ (Rosi) and 2 × 10^7^ (HCT116) cells, respectively) was used. Extracts were pre-cleared for 2 h at 4 °C (with rotation) by incubation with 150 μl of 1:1 PBS:protein-G sepharose beads (GE Healthcare, 17-0618-01). After pre-clearing, the lysates were centrifuged at 10,000 r.p.m. for 10 min at 4 °C. The supernatants were transferred to new tubes and pre-incubated (with rotation) with anti-β-catenin (Abcam, ab6302), anti-CAV1 (Cell Signaling, 3238), anti-PTEN (Cell Signaling, 9559) or control IgG (Cell Signaling, 2729) antibodies at 4 °C for 3 h (all at 1/50 antibody:extract). Following pre-incubation, 150 μl PBS:protein G sepharose (2:1) was added to each antibody-extract mixture and left to rotate at 4 °C overnight.

The next morning, the immune complexes were collected by centrifugation at 14,000 r.p.m. for 10 min at 4 °C. The IP supernatants were removed and the beads were washed four times with IP buffer, with centrifugation at 10,000 r.p.m. for 10 s between each wash. The beads were then washed four times with EDTA buffer (50 mM Tris (pH 8), 150 mM NaCl, 1 mM EDTA, 1% (v/v) Triton X-100). Following the last wash, the immunoprecipitated proteins were solubilized in SDS sample buffer and boiled for 10 min. Samples were then resolved by SDS–PAGE. Trueblot anti-rabbit IgG HRP (18-8816-33, Rockland Immunochemicals) was used as a secondary antibody for western blotting in Fig. 2b,c and Supplementary Fig. 2a.

### Nuclear β-catenin quantification

Quantification of all images was performed using the ImageJ imaging software. Briefly, using the Threshold function, all images were equally calibrated to either control GFP or si Scramble. Once a black and white image was obtained after processing through the Threshold function, a merge with 4,6-diamidino-2-phenylindole and other fluorescent channels (that is, GFP or red fluorescent protein) was generated, after which point we manually counted only the cells that were positive for GFP or red fluorescent protein (depending on the construct) and displayed a nuclear signal.

### Subcellular fractionation

Hs944T cells transfected with complementary DNAs encoding either GFP or PTEN were separated into nuclear and cytoplasmic fractions. Cells were lysed with cytoplasmic extraction buffer (10 mM HEPES pH 7.9, 10 mM KCl, 0.1 mM EDTA, 1.5 mM MgCl_2_, 1 mM dithiothreitol, 0.2% Nonidet P-40, 1 mM NaF, 1 mM Na_3_VO_4_ and protease inhibitor cocktail), while rotating on a rocker rotator at 4 °C for 15 min. The cells were then centrifuged at 14,000 r.p.m. at 4 °C for 5 min and the resulting supernatant (cytoplasmic fraction) was collected. The pellet was resuspended in nuclear extraction buffer (20 mM HEPES pH7.9, 420 mM NaCl, 0.1 mM EDTA pH8, 1.5 mM MgCl_2_, 1 mM dithiothreitol, 0.2%Triton X-100, 1 mM NaF, 1 mM Na_3_VO_4_ and protease inhibitor cocktail) and incubated at room temperature for 10 min, after which it was centrifuged at 14,000 r.p.m. at 4 °C for 5 min. The resultant supernatant (nuclear fraction) was removed from the pellet (cytoskeleton) and the purity of each fraction was assessed by immunoblotting with antibodies to α-tubulin and lamin B1, respectively.

### LiCl and LY294002 treatment

After transfection, cells were treated with 40 mM LiCl for 1 h (ref. 33). Similarly, cells were treated with the PI3K inhibitor LY294002 (Calbiochem, 440202), with 50 μM before lysis for 1 h (ref. 57).

### Pull-down experiments

Five hundred micrograms of whole-cell lysates were incubated with 20 μl of β-catenin–GST beads in PBS supplemented with Protease inhibitors for 3 h at 4 °C under slight agitation. Beads were then collected by centrifugation at 10,000 r.p.m. for 10 min at 4 °C and washed 4 times with 10 mM Tris (pH8), 150 mM NaCl, 1%(v/v) Triton X-100, 60 nM Octyl β-D-glucopyranoside (Sigma, O8001). Samples were resolved in 12% SDS–PAGE gel.

### Luciferase assay

Cells were transiently transfected in 12-well plates with Magnetofectamine (OZ Biosciences) following the manufacturer's specifications. Briefly, cells were transfected with 1.25 μg of total plasmid DNA. As control cells were also co-transfected with thymidine kinase::*Renilla* luciferase^58^. Luciferase activity was determined 48 h post transfection using a MicroLumat PLUS LB 96 V luminometer (Berthold Technologies) and normalized to *Renilla* activity. Briefly, cells were transfected with 750 ng of either *CMV::CAV1-GFP* (#1040), *CMV::PTEN-GFP*^50^ (1031), *CMV::β-catenin*^33^ (997) or *CMV::GFP* (1085) and 350 ng of *CDKN2A::Luciferase* or *MITF::Luciferase*^32^ (778 and 961) and TOP (390) or FOP (391) constructs. The TOP activity is normalized with FOP. Cells were co-transfected with thymidine kinase::*Renilla* luciferase construct as a control. Luciferase activity was determined 48 h post transfection and normalized to *Renilla* luciferase. Statistical analysis was performed using Prism v5.0.

### siRNA knockdown

siRNA targeting human *Caveolin 1* (39) *β-catenin* (35) were purchased from Dharmacon as a SMART pool mix of four sequences and *PTEN* (38) from Santa Cruz Biotechnologies as a mix of three sequences. *Caveolin-1*: 5′-GCA AAU ACG UAG ACU CGG A-3′, 5′-AUU AAG AGC UUC CUG AUU G-3′, 5′-GCA GUU GUA CCA UGC AUU A-3′ and 5′-CUA AAC ACC UCA ACG AUG A-3′. *β-Catenin*: 5′-GAA CGC AGC AGC AGU UUG U-3′, 5′-CAG CUG GCC UGG UUU GAU A-3′, 5′-GCA AGU AGC UGA UAU UGA C-3′ and 5′-GAU CUU AGC UUA UGG CAA U-3′. si Scrambled, with no known human targets, was purchased from Invitrogen as a mix. Briefly, cells were transfected with 50 pmol of siPTEN, siCAV1, siβ-catenin or siScrambled (siScr) and were assayed for Luciferase activity or protein content 48 h post transfection.

### Patients and tumour material

The first set of anonymized human melanoma samples was genomically profiled^59^. The current analyses did not contain patient-specific information and did not need patients' consent. The second human melanoma set included specimens (101 paraffin-embedded samples) from 15 primary melanomas (9 superficial spreading melanomas, 3 nodular melanomas, 1 melanoma, 2 lentigo malignant melanomas), 16 lymph node metastases, 65 cutaneous metastasis and 5 visceral metastases from the pathology archive of the Radboud University Nijmegen Medical Center. Tissues were obtained, stored and used in an anonymized manner according to the code for proper secondary use of human tissue that is published by the Dutch Council of the Federation of Medical Scientific Societies (www.federa.org/codes-conduct) and approved by the institutional review board of the Radboud University Nijmegen Medical Center. Pathological and genomic data were obtained from paraffin-embedded tumour tissue.

### Immunohistochemistry of human tissues

Staining for p16 and CAV1 expression was performed on a Discovery XT Autostainer (Ventana Medical System). All solutions used for automated immunohistochemistry were from Ventana Medical System, unless otherwise specified. Tissue section (4 μm) underwent de-paraffinization with the EZ PREP solution, heat-induced epitope retrieval with Cell Conditioning solution CC1, pH 8.0, at standard condition (60 min at 95 °C). Pre-diluted mouse monoclonal anti-p16 (clone E6H4, CINTec Roche) or polyclonal anti-CAV1 (1/200, 3238, Cell Signalling) diluted in antibody diluent were incubated for 32 min at 37 °C, then followed by the detection kit (Omnimap anti-Mouse HRP, Ref 760–4310) and developed with 3,3′-Diaminobenzidine (DAB). A negative control was performed by the omission of the primary antibody. Slides were counterstained with haematoxylin for 4 min , blued with Bluing Reagent for 4 min , removed from the autostainer, washed in warm soapy water, dehydrated through graded alcohols, cleared in xylene and mounted with Permount. Immunostaining for PTEN was performed manually. Following de-waxing and rehydration through xylene and graded alcohol baths, quenching of endogenous peroxidase (6% hydrogen peroxide in PBS, 30 min, RT), a microwave antigen-retrieval step was performed (TRIS/EDTA pH 9, 10 min at 95 °C and then cooled for 1 h). Each section was incubated with mouse monoclonal anti-PTEN antibody (1/100, clone 6H2.1, Dako) overnight at 4 °C. Following three 10-min washes with PBS, tissues were incubated with powervision poly-HRP-GAM/R/R IgG (Immunologic) and immunoreactivity visualized with PowerDAB (Immunologic), counterstained with haematoxylin and mounted with Permount. Slides were scanned with a ScanScope AT Turbo scanner (Aperio, Leica Biosystems) at × 20 magnification. Vascular endothelium served as an internal positive control for PTEN expression and breast carcinoma as an external positive control. Cytoplasmic and nuclear PTEN and p16 expression was scored as positive immunostaining. CAV1 staining included membranous and/or cytoplasmic expression. Two events occur independently when the % PTEN negative (36/(101–3)) times the % NRAS mutant (11/(101–3)) equals to the % PTEN negative and % NRAS mutant (3/101).

### Detection of NRAS mutations from human tissue

DNA was extracted from 20-μm-thick paraffin-embedded sections using NucleoSpin Extract II (Macherey-Nagel, 740590250) according to the manufacturer's instructions and was amplified by PCR. Allele-specific PCR of the *NRAS* gene was performed using a 5′ WT PCR primer (LL1827, 5′-CAT ACT GGA TAC AGC TGG AC-3′) and a mutated PCR primer corresponding to the NRAS^Q61K^ mutation (LL1828, 5′-CAT ACT GGA TAC AGC TGG GA-3′). The reverse primer (LL1800, 5′-TGA CTT GCT ATT ATT GAT GG-3′) was used for all the PCR reactions^60^. The PCR mixture contained Expand High fidelity buffer, 200 mM of each dNTP (dNTP mix, Finnzyme, F560XL), 50 pM of each primer, 2.6 U of Expand High fidelity (Roche, 11732650001) and 100 ng of DNA. PCR was performed for 38 cycles of 30 s at 94 °C, 90 s at 56 °C and 30 s at 72 °C. Samples were incubated for 10 min at 94 °C before the cycles. The *NRAS* gene in DNA extracted from tumours was also sequenced following PCR amplification. The primer sequences were 5′-GTT ATA GAT GGT GAA ACC TG-3′ (LL1901; forward) and 5′-GAG GTT AAT ATC CGC AAA TGA CTT-3′ (LL1918; reverse). The *NRAS* gene exon 3 sequences were analysed by direct DNA sequencing according to the Sanger technology.

### Transgenic mice and tumour collection

The transgenic *Tyr::N-RAS*^*Q61K*^*/°* mouse line was described previously^3^. Floxed PTEN mice were provided by H. Wu (UCLA, Los Angeles, CA, USA) and were obtained from F. Beermann (EPFL, Lausanne, Switzerland). The characterization of the PTEN flox mice^61^,^62^ and Tyr::Cre mice^55^ has been reported previously. All mice were backcrossed onto a C57BL/6 background for more than ten generations. Mice were maintained in the specific pathogen-free mouse colony at the Institut Curie, in line with French and European Union law. Ethical authorization number is P2.LL.029.07. Floxed PTEN heterozygous mice were crossed with Tyr::Cre and Tyr::NRAS^Q61K^/° to generate *Tyr::NRAS*^*Q61K*^*/°;Tyr::Cre; PTEN*^*f/+*^ (*NRAS-ΔPTEN* mice), *Tyr::NRAS*^*Q61K*^*/°; PTEN*^*f/+*^ (*NRAS* mice) and *Tyr::NRAS*^*Q61K*^*/°;PTEN*^*f/f*^ (*NRAS* mice). Mice were genotyped by PCR using DNA extracted from tails. The mice were evaluated weekly for tumour appearance and progression. Once tumours were 1 cm across, the mice were killed and autopsied. Some mice were also killed because of poor health. Tumour samples were fixed in 4% PFA and paraffin-embedded for histological analysis and immunostaining. When sufficient tumour tissue was available, samples were frozen for subsequent western blot analysis.

### Histology and immunostaining of mouse tissues

Mouse melanomas were collected, rinsed in cold PBS and fixed in 4% PFA at 4 °C O/N. Samples were dehydrated, embedded in paraffin wax and sectioned into 5-μm-thick transverse sections. Paraffin-embedded sections were stained with haematoxylin and eosin, and examined by light microscopy. For immunostaining, sections were deparaffinized, rinsed in Tween Buffer saline (TBS; Tris 20mM pH 7,6, NaCl 150mM and Tween 20 0.1%), boiled for 20 min in 10 mM sodium citrate and treated overnight at 4 °C in TBST (TBS/0.1% Tween-20) containing 5% normal goat serum with antibodies against S100 (Dako, Z0311, dilution 1/100). AEC (Sigma-Aldrich, A6926) was used to reveal bound antibody according to the manufacturer's instructions. All sections were counterstained with haematoxylin. Ki-67 (Nova-Costra, NCL-Ki67p) and gp84 (ECAD antibody) antibodies were both produced in rabbit.

### miRNA overexpression

Cells were transfected with 100 nM of miRNA mimics, hsa-miR-203 (miRIDIAN microRNA mimic; Dharmacon c-300562-03-0002) and hsa-miR-199a-5p (miRIDIAN microRNA mimic; Dharmacon c-300533-03-0005). Forty-eight hours post transfection, cells were lysed for mRNA and protein contents, and analysed.

## Additional information

**How to cite this article:** Conde-Perez, A. *et al.* A caveolin-dependent and PI3K/AKT-independent role of PTEN in β-catenin transcriptional activity. *Nat. Commun.* 6:8093 doi: 10.1038/ncomms9093 (2015).

## Supplementary Material

Supplementary Information Supplementary Figures 1-9 and Supplementary Table 1.

Supplementary Data 1 CTNNB1 interactome

Supplementary Data 2 PTEN interactome

Supplementary Data 3 Common list for PTEN and CTNNB1

Supplementary Data 4 miRnome of murine *NRAS* and *NRAS-ΔPTEN* tumors

## References

[b1] Dhomen N. *et al.* Oncogenic Braf induces melanocyte senescence and melanoma in mice. Cancer Cell 15, 294–303 (2009). 19345328 10.1016/j.ccr.2009.02.022

[b2] Michaloglou C. *et al.* BRAFE600-associated senescence-like cell cycle arrest of human naevi. Nature 436, 720–724 (2005). 16079850 10.1038/nature03890

[b3] Ackermann J. *et al.* Metastasizing melanoma formation caused by expression of activated N-RasQ61K on an INK4a-deficient background. Cancer Res. 65, 4005–4011 (2005). 15899789 10.1158/0008-5472.CAN-04-2970

[b4] Barradas M. *et al.* Histone demethylase JMJD3 contributes to epigenetic control of INK4a/ARF by oncogenic RAS. Genes Dev. 23, 1177–1182 (2009). 19451218 10.1101/gad.511109 PMC2685533

[b5] Shakhova O. *et al.* Sox10 promotes the formation and maintenance of giant congenital naevi and melanoma. Nat. Cell Biol. 14, 882–890 (2012). 22772081 10.1038/ncb2535

[b6] Gray-Schopfer V. C. *et al.* Cellular senescence in naevi and immortalisation in melanoma: a role for p16? Br. J. Cancer 95, 496–505 (2006). 16880792 10.1038/sj.bjc.6603283 PMC2360676

[b7] Zhou X. P. *et al.* Epigenetic PTEN silencing in malignant melanomas without PTEN mutation. Am. J Pathol. 157, 1123–1128 (2000). 11021816 10.1016/S0002-9440(10)64627-5 PMC1850161

[b8] Whiteman D. C. *et al.* Nuclear PTEN expression and clinicopathologic features in a population-based series of primary cutaneous melanoma. Int. J. Cancer 99, 63–67 (2002). 11948493 10.1002/ijc.10294

[b9] Wu H., Goel V. & Haluska F. G. PTEN signaling pathways in melanoma. Oncogene 22, 3113–3122 (2003). 12789288 10.1038/sj.onc.1206451

[b10] Goel V. K., Lazar A. J., Warneke C. L., Redston M. S. & Haluska F. G. Examination of mutations in BRAF, NRAS, and PTEN in primary cutaneous melanoma. J. Invest. Dermatol. 126, 154–160 (2006). 16417231 10.1038/sj.jid.5700026

[b11] Wakisaka T. & Sou S. [A case of recurrent ovarian cancer successfully treated with etoposide]. Gan To Kagaku Ryoho 15, 1795–1798 (1988). 3369875

[b12] Tsao H., Mihm M. C. Jr. & Sheehan C. PTEN expression in normal skin, acquired melanocytic nevi, and cutaneous melanoma. J. Am. Acad. Dermatol. 49, 865–872 (2003). 14576666 10.1016/s0190-9622(03)02473-3

[b13] Inoue-Narita T. *et al.* Pten deficiency in melanocytes results in resistance to hair graying and susceptibility to carcinogen-induced melanomagenesis. Cancer Res. 68, 5760–5768 (2008). 18632629 10.1158/0008-5472.CAN-08-0889

[b14] Song M. S. *et al.* Nuclear PTEN regulates the APC-CDH1 tumor-suppressive complex in a phosphatase-independent manner. Cell 144, 187–199 (2011). 21241890 10.1016/j.cell.2010.12.020 PMC3249980

[b15] Kennedy A. L. *et al.* Activation of the PIK3CA/AKT pathway suppresses senescence induced by an activated RAS oncogene to promote tumorigenesis. Mol. Cell 42, 36–49 (2011). 21474066 10.1016/j.molcel.2011.02.020 PMC3145340

[b16] Vredeveld L. C. *et al.* Abrogation of BRAFV600E-induced senescence by PI3K pathway activation contributes to melanomagenesis. Genes Dev. 26, 1055–1069 (2012). 22549727 10.1101/gad.187252.112 PMC3360561

[b17] Dankort D. *et al.* Braf(V600E) cooperates with Pten loss to induce metastatic melanoma. Nat. Genet. 41, 544–552 (2009). 19282848 10.1038/ng.356 PMC2705918

[b18] Conde-Perez A. & Larue L. PTEN and melanomagenesis. Future Oncol. 8, 1109–1120 (2012). 23030486 10.2217/fon.12.106

[b19] Caselli A., Mazzinghi B., Camici G., Manao G. & Ramponi G. Some protein tyrosine phosphatases target in part to lipid rafts and interact with caveolin-1. Biochem. Biophys. Res. Commun. 296, 692–697 (2002). 12176037 10.1016/s0006-291x(02)00928-2

[b20] Chalecka-Franaszek E. & Chuang D. M. Lithium activates the serine/threonine kinase Akt-1 and suppresses glutamate-induced inhibition of Akt-1 activity in neurons. Proc. Natl Acad. Sci. USA 96, 8745–8750 (1999). 10411946 10.1073/pnas.96.15.8745 PMC17587

[b21] Yuan G. *et al.* WNT6 is a novel target gene of caveolin-1 promoting chemoresistance to epirubicin in human gastric cancer cells. Oncogene 32, 375–387 (2013). 22370641 10.1038/onc.2012.40

[b22] Xia H. *et al.* Pathologic caveolin-1 regulation of PTEN in idiopathic pulmonary fibrosis. Am. J. Pathol. 176, 2626–2637 (2010). 20395445 10.2353/ajpath.2010.091117 PMC2877826

[b23] Kronstein R. *et al.* Caveolin-1 opens endothelial cell junctions by targeting catenins. Cardiovasc. Res. 93, 130–140 (2012). 21960684 10.1093/cvr/cvr256

[b24] Wypijewski K. J. *et al.* Identification of caveolar resident proteins in ventricular myocytes using a quantitative proteomic approach: dynamic changes in caveolar composition following adrenoceptor activation. Mol. Cell Proteomics 14, 596–608 (2015). 25561500 10.1074/mcp.M114.038570 PMC4349980

[b25] Orlichenko L. *et al.* Caveolae mediate growth factor-induced disassembly of adherens junctions to support tumor cell dissociation. Mol. Biol. Cell 20, 4140–4152 (2009). 19641024 10.1091/mbc.E08-10-1043 PMC2754928

[b26] Morali O. G. *et al.* IGF-II induces rapid beta-catenin relocation to the nucleus during epithelium to mesenchyme transition. Oncogene 20, 4942–4950 (2001). 11526479 10.1038/sj.onc.1204660

[b27] Miao L. *et al.* miR-203 inhibits tumor cell migration and invasion via caveolin-1 in pancreatic cancer cells. Oncol. Lett. 7, 658–662 (2014). 24520289 10.3892/ol.2014.1807 PMC3919932

[b28] Orom U. A. *et al.* MicroRNA-203 regulates caveolin-1 in breast tissue during caloric restriction. Cell Cycle 11, 1291–1295 (2012). 22421148 10.4161/cc.19704 PMC3350875

[b29] Lino Cardenas C. L. *et al.* miR-199a-5p Is upregulated during fibrogenic response to tissue injury and mediates TGFbeta-induced lung fibroblast activation by targeting caveolin-1. PLoS Genet. 9, e1003291 (2013). 23459460 10.1371/journal.pgen.1003291 PMC3573122

[b30] Bucheit A. D. *et al.* Complete loss of PTEN protein expression correlates with shorter time to brain metastasis and survival in stage IIIB/C melanoma patients with BRAFV600 mutations. Clin. Cancer Res. 20, 5527–5536 (2014). 25165098 10.1158/1078-0432.CCR-14-1027 PMC4216767

[b31] Sviderskaya E. V. *et al.* p16(Ink4a) in melanocyte senescence and differentiation. J. Natl Cancer Inst. 94, 446–454 (2002). 11904317 10.1093/jnci/94.6.446

[b32] Delmas V. *et al.* Beta-catenin induces immortalization of melanocytes by suppressing p16INK4a expression and cooperates with N-Ras in melanoma development. Genes Dev. 21, 2923–2935 (2007). 18006687 10.1101/gad.450107 PMC2049194

[b33] Gallagher S. J. *et al.* Beta-catenin inhibits melanocyte migration but induces melanoma metastasis. Oncogene 32, 2230–2238 (2013). 22665063 10.1038/onc.2012.229 PMC3637425

[b34] Chien A. J. *et al.* Activated Wnt/beta-catenin signaling in melanoma is associated with decreased proliferation in patient tumors and a murine melanoma model. Proc. Natl Acad. Sci. USA 106, 1193–1198 (2009). 19144919 10.1073/pnas.0811902106 PMC2626610

[b35] Domingues M. J. *et al.* beta-catenin inhibitor ICAT modulates the invasive motility of melanoma cells. Cancer Res. 74, 1983–1995 (2014). 24514042 10.1158/0008-5472.CAN-13-0920

[b36] Arozarena I. *et al.* In melanoma, beta-catenin is a suppressor of invasion. Oncogene 30, 4531–4543 (2011). 21577209 10.1038/onc.2011.162 PMC3160497

[b37] Sinnberg T. *et al.* Beta-catenin signaling increases during melanoma progression and promotes tumor cell survival and chemoresistance. PLoS ONE 6, e23429 (2011). 21858114 10.1371/journal.pone.0023429 PMC3157382

[b38] James R. G. *et al.* Protein kinase PKN1 represses Wnt/beta-catenin signaling in human melanoma cells. J. Biol. Chem. 288, 34658–34670 (2013). 24114839 10.1074/jbc.M113.500314 PMC3843078

[b39] Lobos-Gonzalez L. *et al.* E-cadherin determines Caveolin-1 tumor suppression or metastasis enhancing function in melanoma cells. Pigment Cell Melanoma Res. 26, 555–570 (2013). 23470013 10.1111/pcmr.12085 PMC3695072

[b40] Felicetti F. *et al.* Caveolin-1 tumor-promoting role in human melanoma. Int. J. Cancer 125, 1514–1522 (2009). 19521982 10.1002/ijc.24451 PMC2805039

[b41] Burgermeister E., Liscovitch M., Rocken C., Schmid R. M. & Ebert M. P. Caveats of caveolin-1 in cancer progression. Cancer Lett. 268, 187–201 (2008). 18482795 10.1016/j.canlet.2008.03.055

[b42] Yamamoto H., Sakane H., Michiue T. & Kikuchi A. Wnt3a and Dkk1 regulate distinct internalization pathways of LRP6 to tune the activation of beta-catenin signaling. Dev. Cell 15, 37–48 (2008). 18606139 10.1016/j.devcel.2008.04.015

[b43] Tsao H., Zhang X., Fowlkes K. & Haluska F. G. Relative reciprocity of NRAS and PTEN/MMAC1 alterations in cutaneous melanoma cell lines. Cancer Res. 60, 1800–1804 (2000). 10766161

[b44] Alexaki V. I. *et al.* GLI2-mediated melanoma invasion and metastasis. J. Natl Cancer Inst. 102, 1148–1159 (2010). 20660365 10.1093/jnci/djq257 PMC2914763

[b45] Hamai A. *et al.* ICAM-1 has a critical role in the regulation of metastatic melanoma tumor susceptibility to CTL lysis by interfering with PI3K/AKT pathway. Cancer Res. 68, 9854–9864 (2008). 19047166 10.1158/0008-5472.CAN-08-0719

[b46] Hoeflich K. P. *et al.* Requirement for glycogen synthase kinase-3beta in cell survival and NF-kappaB activation. Nature 406, 86–90 (2000). 10894547 10.1038/35017574

[b47] Halaban R. *et al.* PLX4032, a selective BRAF(V600E) kinase inhibitor, activates the ERK pathway and enhances cell migration and proliferation of BRAF melanoma cells. Pigment Cell Melanoma Res. 23, 190–200 (2010). 20149136 10.1111/j.1755-148X.2010.00685.x PMC2848976

[b48] Moore R. *et al.* Involvement of cadherins 7 and 20 in mouse embryogenesis and melanocyte transformation. Oncogene 23, 6726–6735 (2004). 15273735 10.1038/sj.onc.1207675

[b49] Tibarewal P. *et al.* PTEN protein phosphatase activity correlates with control of gene expression and invasion, a tumor-suppressing phenotype, but not with AKT activity. Sci. Signal 5, ra18 (2012). 22375056 10.1126/scisignal.2002138

[b50] Liu F. *et al.* PTEN enters the nucleus by diffusion. J Cell Biochem 96, 221–234 (2005). 16088943 10.1002/jcb.20525

[b51] Samuels Y. *et al.* High frequency of mutations of the PIK3CA gene in human cancers. Science 304, 554 (2004). 15016963 10.1126/science.1096502

[b52] Van de Craen M. *et al.* Proteolytic cleavage of beta-catenin by caspases: an *in vitro* analysis. FEBS Lett. 458, 167–170 (1999). 10481058 10.1016/s0014-5793(99)01153-9

[b53] Gallagher S. J. *et al.* General strategy to analyse melanoma in mice. Pigment Cell Melanoma Res. 24, 987–988 (2011). 21955491 10.1111/j.1755-148X.2011.00907.x

[b54] MacKenzie M. A., Jordan S. A., Budd P. S. & Jackson I. J. Activation of the receptor tyrosine kinase Kit is required for the proliferation of melanoblasts in the mouse embryo. Dev. Biol. 192, 99–107 (1997). 9405100 10.1006/dbio.1997.8738

[b55] Delmas V., Martinozzi S., Bourgeois Y., Holzenberger M. & Larue L. Cre-mediated recombination in the skin melanocyte lineage. Genesis 36, 73–80 (2003). 12820167 10.1002/gene.10197

[b56] Capozza F. *et al.* Muscle-specific interaction of caveolin isoforms: differential complex formation between caveolins in fibroblastic vs. muscle cells. Am. J. Physiol. Cell Physiol. 288, C677–C691 (2005). 15548572 10.1152/ajpcell.00232.2004

[b57] Aziz S. A. *et al.* Phosphatidylinositol-3-kinase as a therapeutic target in melanoma. Clin. Cancer Res. 15, 3029–3036 (2009). 19383818 10.1158/1078-0432.CCR-08-2768 PMC4431617

[b58] Berlin I. *et al.* Phosphorylation of BRN2 modulates its interaction with the Pax3 promoter to control melanocyte migration and proliferation. Mol. Cell Biol. 32, 1237–1247 (2012). 22290434 10.1128/MCB.06257-11 PMC3302439

[b59] Curtin J. A. *et al.* Distinct sets of genetic alterations in melanoma. N. Engl. J. Med. 353, 2135–2147 (2005). 16291983 10.1056/NEJMoa050092

[b60] Linard B. *et al.* A ras-mutated peptide targeted by CTL infiltrating a human melanoma lesion. J. Immunol. 168, 4802–4808 (2002). 11971032 10.4049/jimmunol.168.9.4802

[b61] Groszer M. *et al.* Negative regulation of neural stem/progenitor cell proliferation by the Pten tumor suppressor gene in vivo. Science 294, 2186–2189 (2001). 11691952 10.1126/science.1065518

[b62] Lesche R. *et al.* Cre/loxP-mediated inactivation of the murine Pten tumor suppressor gene. Genesis 32, 148–149 (2002). 11857804 10.1002/gene.10036

