# Supplementary material for: A caveolin-dependent and PI3K/AKT-independent role of PTEN in β-catenin transcriptional activity
Source: Nat Commun. 2015 Aug 26;6:8093. doi: 10.1038/ncomms9093 (PMC4560817; doi:10.1038/ncomms9093)
Supplement: Supplementary Information — Supplementary Figures 1-9 and Supplementary Table 1. [file ncomms9093-s1.pdf]

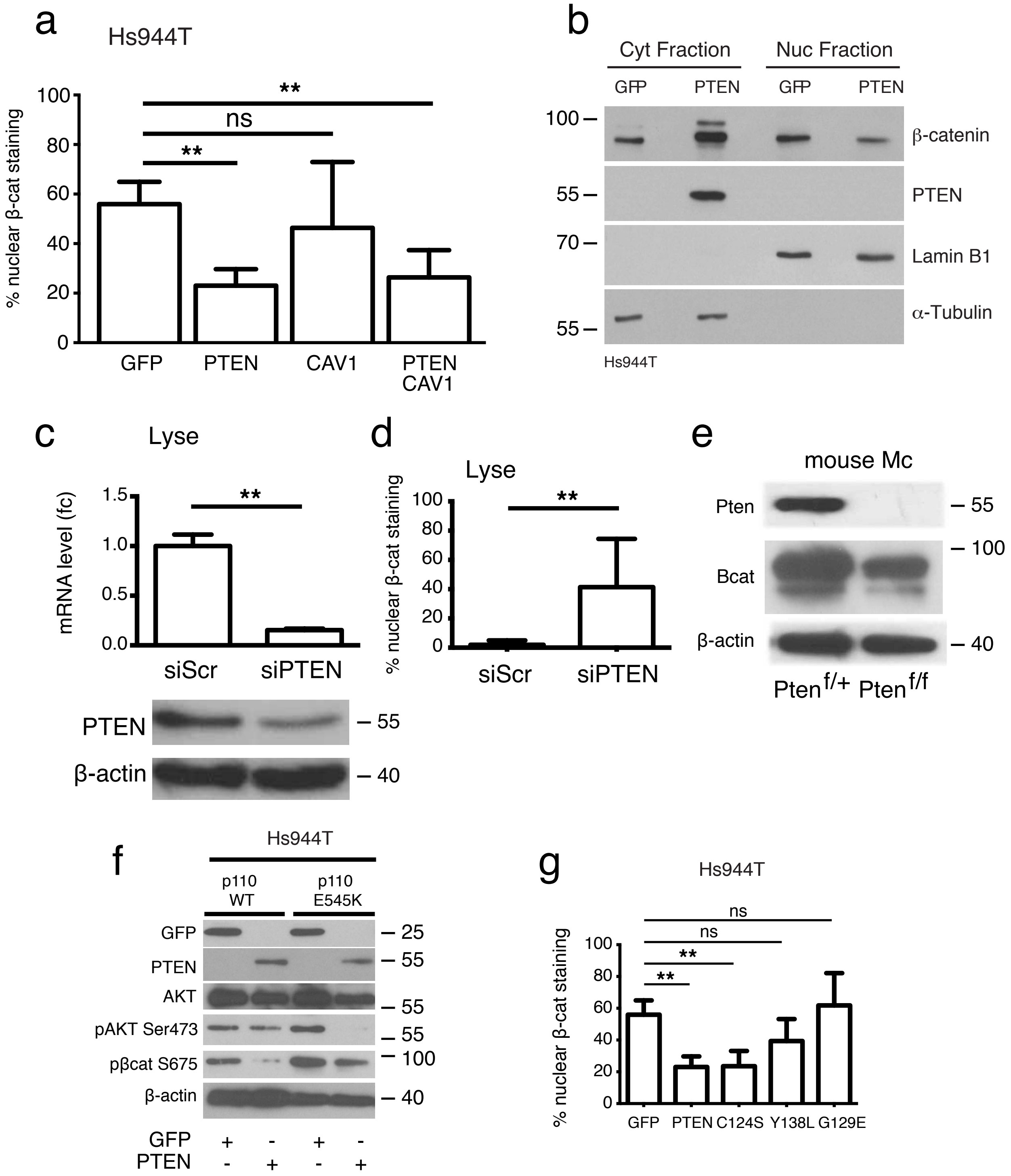

Supplementary Figure 1 : PTEN affects β-catenin nuclear localization

(A) Histogram depicting the percentage of cells with nuclear β-catenin staining. Hs944T cells were transfected with expression vectors for GFP, PTEN, CAV1, or PTEN and CAV1. In total, 85 were counted with positive GFP, 39 for PTEN, 90 for CAV1, and 60 for PTEN and CAV1. (B) Subcellular fractionation of Hs944T cells transfected with either GFP or PTEN were separated into cytoplasmic (Cyt - α-tubulin) and nuclear (Nuc - lamin B1) fractions and the distribution of β-catenin was assessed by western blot analysis. (C) The down regulation of PTEN protein in Lyse cells in the presence of siScr and siPTEN was evaluated by western blot and by q-RT-PCR analysis. We estimated that the amount of PTEN mRNA and protein was reduced by 85% and 60%, respectively. (D) β-catenin nuclear staining quantification of Lyse human melanoma cell line after transfection with siScr or siPTEN. A total of 335 cells were counted for siScr and 62 for siPTEN. (E) Western blot analysis of total cell lysate, showing Pten and β-catenin (Bcat) protein expression in Ptenf/f and Ptenf/+ melanocytes. β-actin is shown as loading control. Western blot analyses was performed once. (F) Immunoblot blot analysis for GFP, PTEN, AKT (total and pSer473), β-catenin pSer675, and β-actin of Hs944T lysates, prior to co-transfected with either empty vector GFP or PTEN and wild-type (WT) or active p110 E545K mutant. Western blot analyses were performed two times with similar results. (G) Histogram depicting the percentage of cells with nuclear β-catenin staining in Hs944T cells transfected with expression vectors for GFP, PTEN-GFP, PTEN-C124S-GFP, PTEN-Y138L-GFP or PTEN-G129E-GFP. In total 85 cells were counted with positive GFP, 39 for PTEN-GFP, 168 for PTEN-C124S-GFP, 40 for PTEN-Y138L-GFP, and 58 for PTEN-G129E-GFP. Error bars represent standard deviation. ** signifies p-value <0.01 and ns >0.05, respectively. Statistical significance was determined by Mann-Whitney test.

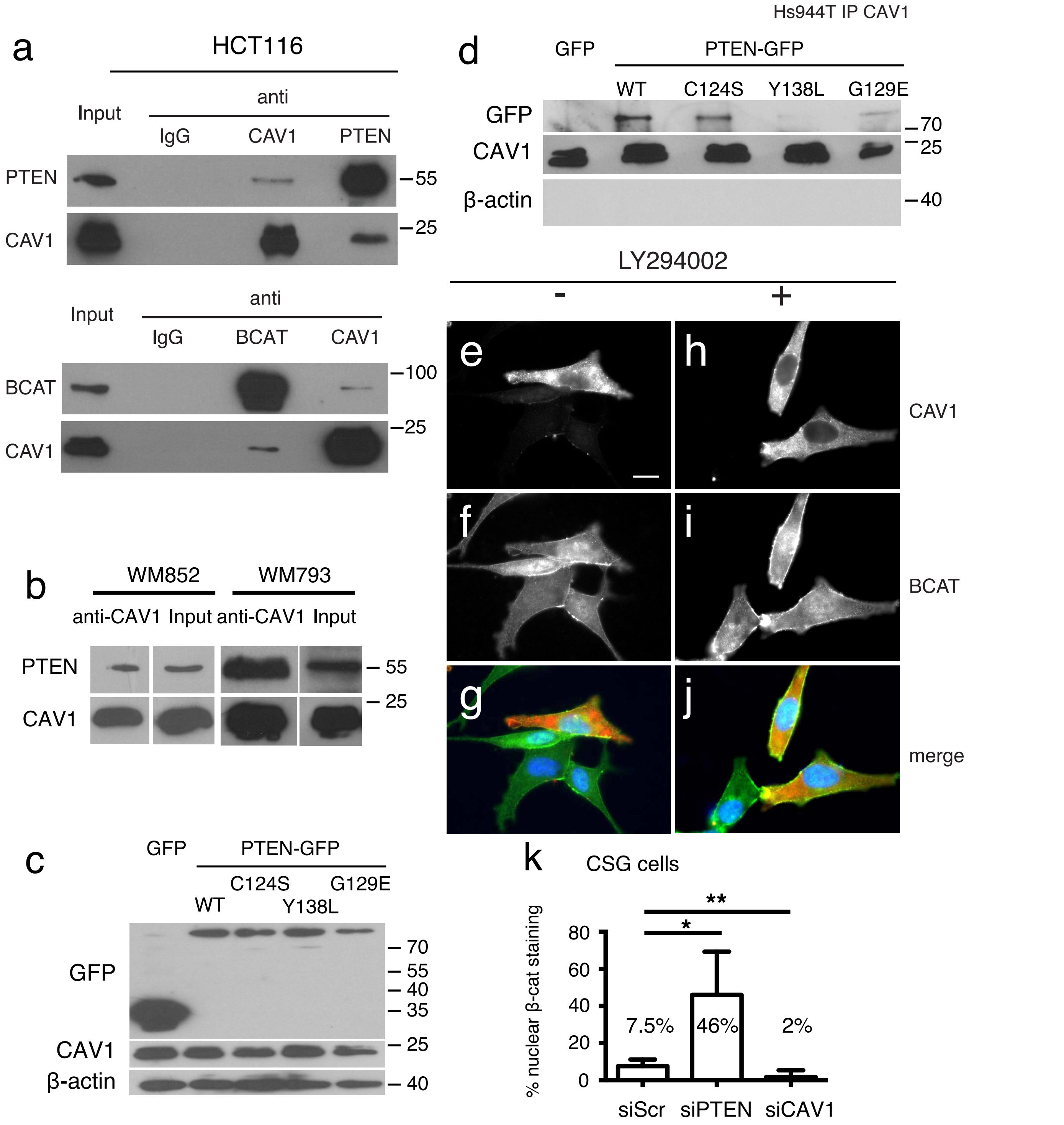

Supplementary Figure 2. CAV1 interacts with PTEN and β-catenin
(A) Interaction of caveolin-1 (CAV1) with PTEN (top) and β-catenin (BCAT; bottom) in HCT116 colon carcinoma cells. Extracts from approximately 2x107 cells were immunoprecipitated with control IgG, anti-BCAT, anti-CAV1 or anti-PTEN antibodies. Immune complexes were resolved by SDS-PAGE and blotted with antibodies to BCAT, CAV1 and PTEN. Total protein input corresponds to 2% of the total protein used for immunoprecipitation. One-tenth of the IP sample was loaded to detect BCAT following IP with BCAT antibodies (and similarly for CAV1 and PTEN). This allowed getting a reasonable intensity for the corresponding signals. For the other lanes, the entire IP samples were loaded. (B) Similarly CAV1-PTEN immunocomplex from engodenous CAV1 and PTEN expression in WM852 and WM793 human melanoma cell lines using an antibody directed against CAV1. Total protein input is shown. (C) Hs944T cells were transfected with expression vectors for GFP, PTEN-GFP, PTEN-C124S-GFP, PTEN-Y138L-GFP or PTEN-G129E-GFP. Whole cell lysates were immunoblotted for GFP, CAV1 and β-actin. (D) Immunoprecipitation of 400 µg lysates from (C) using an anti-CAV1 antibody. Co-immunoprecipitation of PTEN was detected by western blotting with anti-GFP and anti-CAV1 antibodies. (E-J) LY294002 did not affect the nuclear localization of β-catenin in the presence of exogenous CAV1-RFP. Hs944T cells were treated (H-J) or not (E-G) for one hour with LY294002. (E,H) CAV1-RFP and (F,I) β-catenin immunostainings. (G,J) merge: RFP and β-catenin immunostaining were counterstained with DAPI. Note that the dose of LY294002 was subtoxic but affected slightly the shape of the cells. Scale bar = 10µm. (K) Image quantification of nuclear β-catenin positive CSG cells after transfection with negative control siScr, siPTEN, or siCAV1. In total 647 cells were counted for siScr, 437 for siPTEN, and 223 for siCAV1. Error bars represent standard deviation. *, ** signify p-value <0.05 and <0.01 respectively. Statistical significance was determined by Mann-Whitney test.

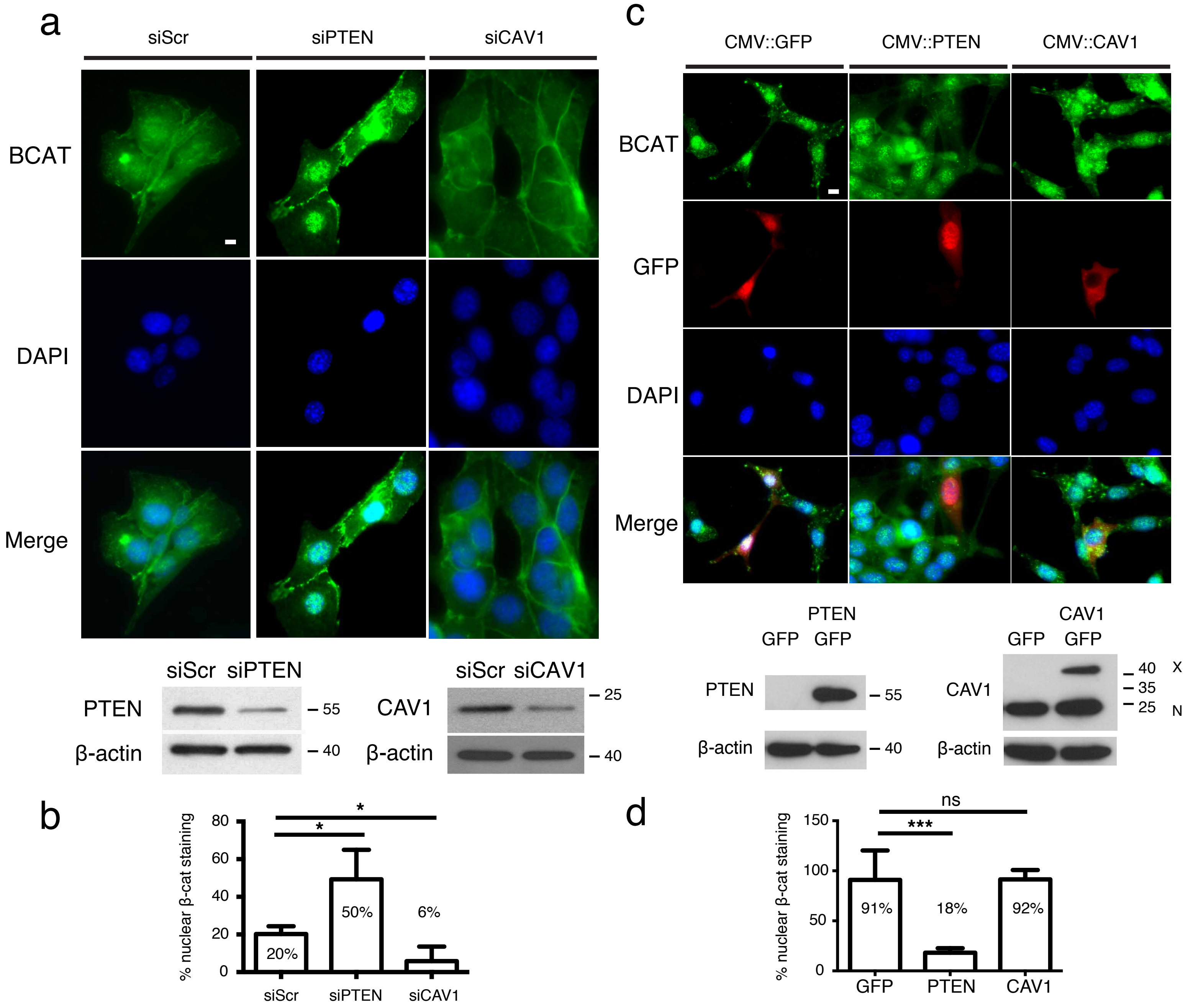

Supplementary Figure 3. PTEN and CAV1 affects the subcellular localization of β-catenin in pancreatic cancer cells

(A) Immunofluorescence using siRNA mediated knockdown of either PTEN or CAV1 in mouse pancreatic cancer cells (KPC) were stained for β-catenin (BCAT) and counterstained with DAPI. Western blot analysis of whole cell protein extracts was used to validate the knockdown of PTEN and CAV1. Scale bar = 25 μm. Immunofluorescence experiments were performed two times with similar results. (B) Image quantification of nuclear β-catenin positive KPC cells after transfection with negative control siScr, siPTEN, or siCAV1. In total 223 cells were counted for siScr, 412 for siPTEN, and 320 for siCAV1. Error bars represent standard deviation. * signifies p-value <0.05. Statistical significance was determined by Mann-Whitney test. (C) Immunofluorescence transfection of PTEN and CAV1 (pseudocolored red) in mouse Pancreatic cancer cells (KPCPTEN2) lacking PTEN protein, stained for β-catenin (BCAT). Western blot analysis of whole cell protein extract was used to validate the overexpression. X = exogenous CAV1, N = endogenous CAV1. Scale bar = 25 μm. Immunofluorescence experiments were performed four times with similar results. (D) Image quantification of nuclear β-catenin positive KPCPTEN2 cells after transfection with negative control GFP, PTEN, or CAV1. In total 34 cells were counted for GFP, 85 for PTEN, and 90 for CAV1. Error bars represent standard deviation. *** signifies p-value <0.001 and ns >0.05, respectively. Statistical significance was determined by Mann-Whitney test.

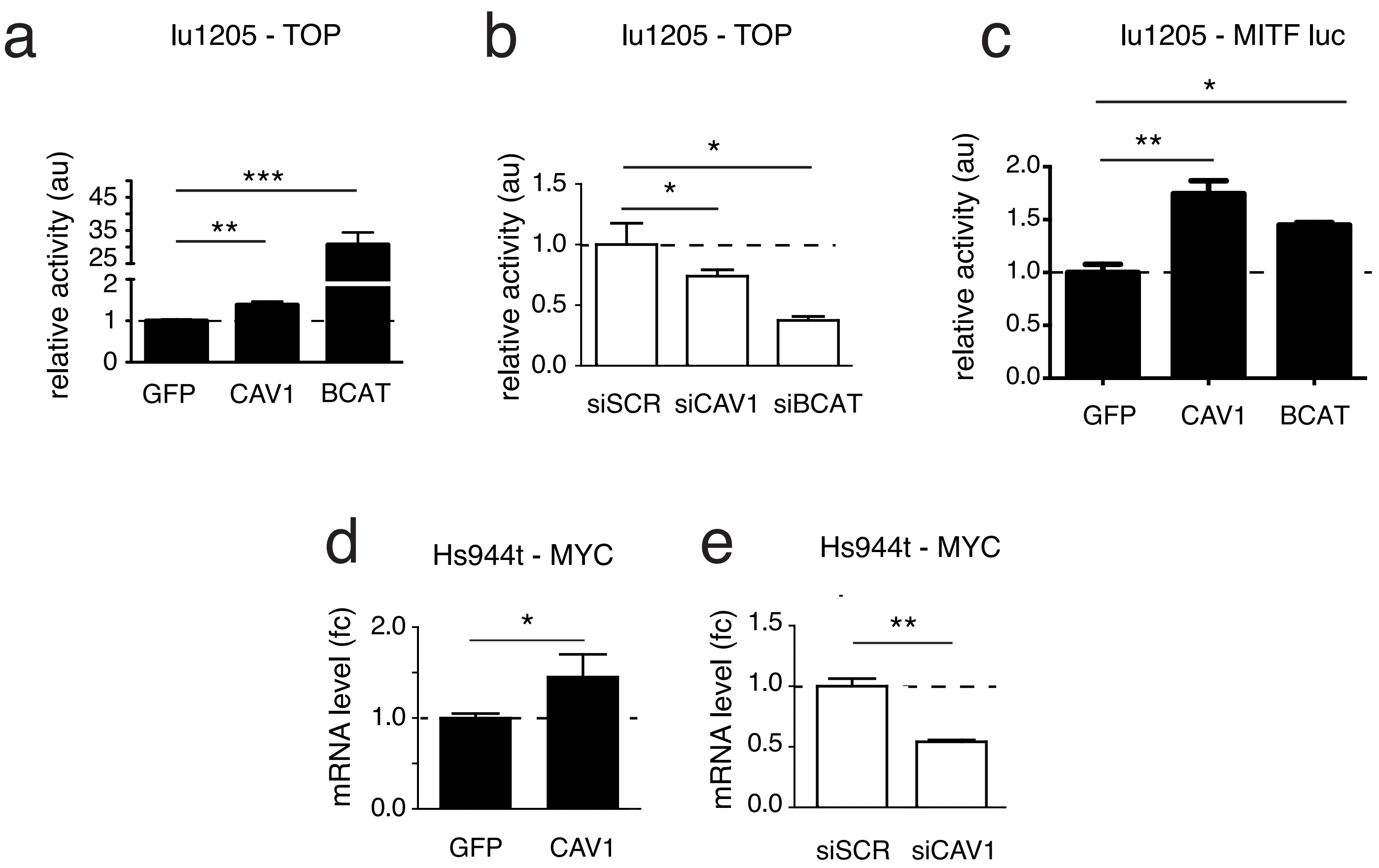

Supplementary Figure 4. CAV1 regulates the level of BCAT targets
(A) TOP-FLASH activity in lu1205 cells after over-expression of GFP, CAV1, and BCAT. (B) Similarly, TOP-FLASH activity was measured in the same cell line 48 hours post transfection of siRNA directed against negative control (siSCR), CAV1 (siCAV1), and β-cat (siBCAT). (C) MITF::luciferase activity was quantified in lu1205 cells after transfection with GFP, CAV1 or BCAT. All TOP-FLASH and MITF::luciferase reporter assays were evaluated in the presence of an internal control (Renilla-luciferase). (D,E) c-MYC mRNA levels as measured by qRT-PCR (fold change), following over-expression or knock-down of CAV1 in Hs944T cell line. Error bars represent standard deviation. *,**, and *** signify p-value <0.05, <0.01, and <0.001, respectively. Statistical significance was determined by Mann-Whitney test. Each experiment was performed in at least biological triplicates.

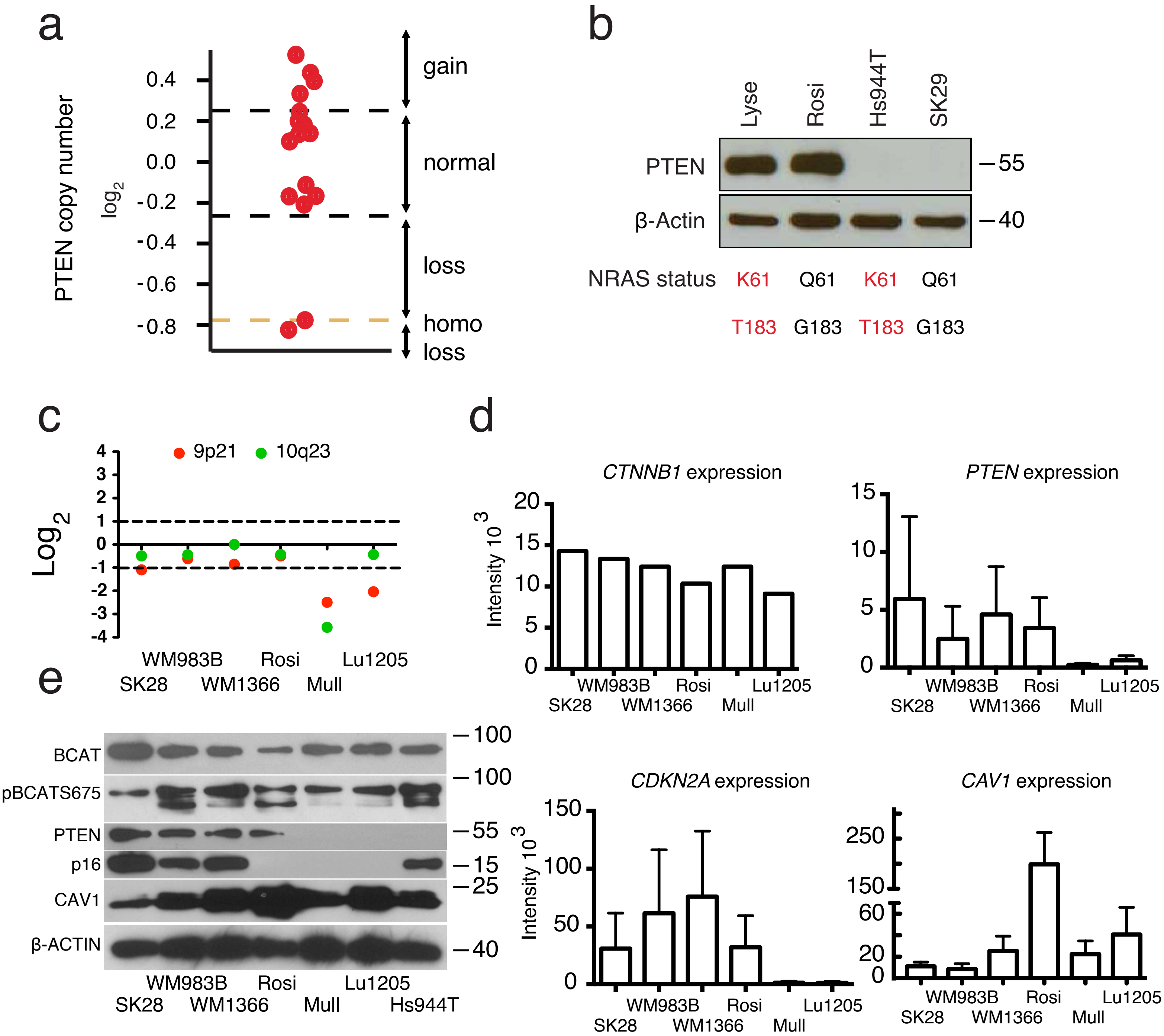

Supplementary Figure 5. Melanoma can be mutated for NRAS and lacking PTEN

(A) A primary library of 105 human melanoma samples was assayed by CGH for PTEN status as well as the presence/absence of NRASQ61K mutation. Sixteen samples were identified as having the NRASQ61K mutation (red full circles). On the y-axis (log2), the PTEN copy number determined by CGH analysis: PTEN was absent from 2 cases out of 16 human melanomas carrying NRASQ61K mutation. (B) The loss of PTEN with coexistent activation of NRAS assayed from four human melanoma cell lines. NRAS mutated cell line carried the K61 mutation validated by allelic specific PCR. Note that another cell line, besides Hs944T, was found to be mutated for NRAS and lacking PTEN (YUDEDE which is NRASQ61H). Western blot analysis for PTEN and β-actin proteins were performed three times with similar results. (C) CGH analysis of p16 (9p21 in red) and PTEN (10q23 in green) status in six different human melanoma cell lines (SK28, WM983B, WM1366, Rosi, Mull, and Lu1205).

(D) Corresponding mRNA expression profiles of SK28, WM983B, WM1366, Rosi, Mull, and Lu1205 cell lines for β-catenin (CTNNB1), PTEN, p16 (CDKN2A), and CAV1 from Affymetrix platform arrays.

(E) Western blot analysis of whole cell extracts of SK28, WM983B, WM1366, Rosi, Mull, Lu1205, Hs944T cell lines for different antibodies (BCAT, PTEN, P16, CAV1, ACTIN). Western blot analysis were performed two times for each antibody with similar results.

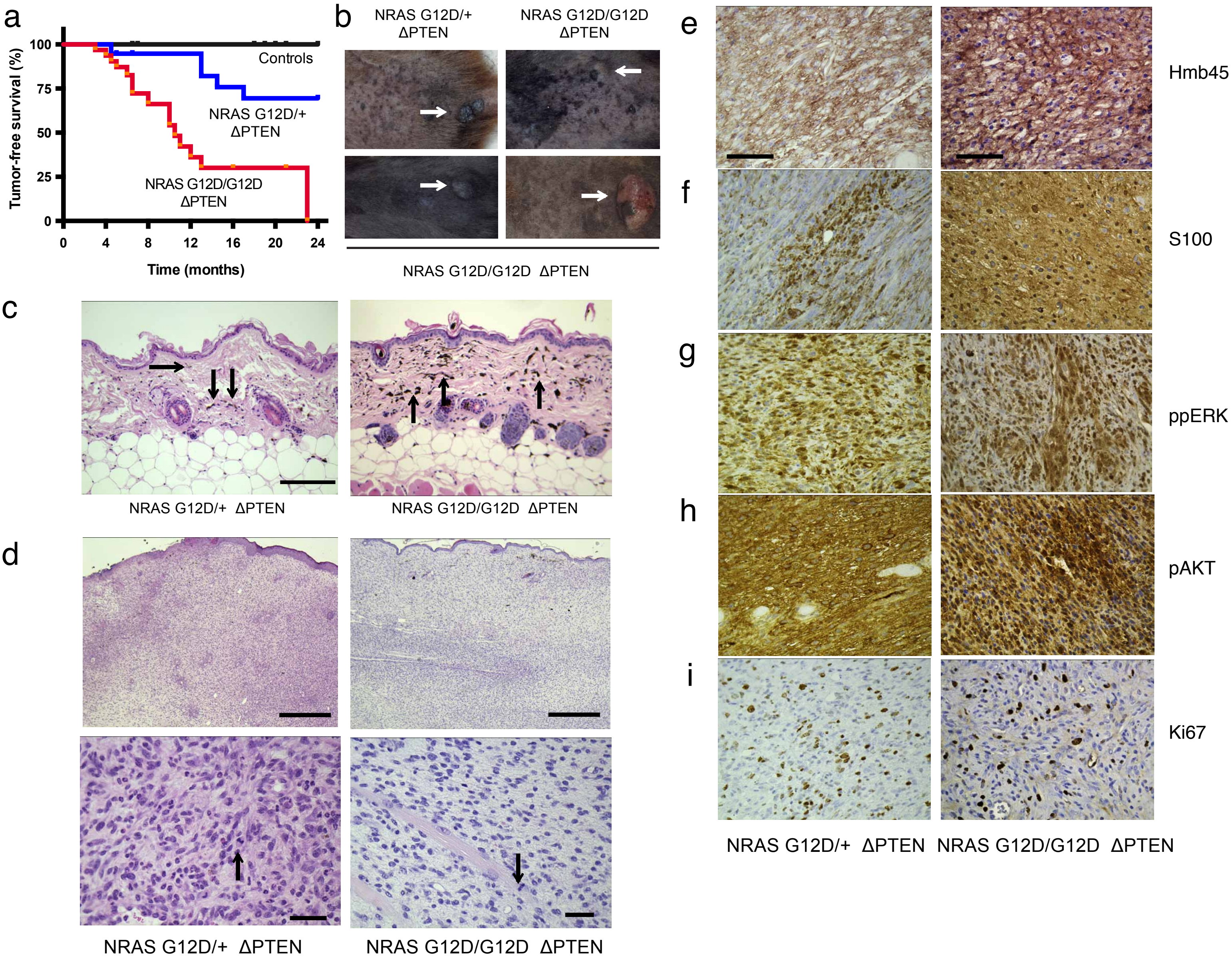

Supplementary Figure 6. Survival curves of mice mutated for NRAS G12D in the presence and absence of PTEN and molecular histology of associated melanoma.
(A) Kaplan-Meier plot showing tumor free survival in months of studied mice.
The experimental groups consisted of tamoxifen-treated Tyr::CreERt2/° ; NrasG12D/+ ; PTENf/+ (NRASG12D/+ - ΔPTEN, n=20), and Tyr::CreERt2/° ; NrasG12D/G12D ; PTENf/+ (NRASG12D/G12D - ΔPTEN, n=32). The control group consisted of tamoxifen treated Tyr::CreERt2/° ; Nras+/+ ; PTENf/+ (n=10), Tyr::CreERt2/° ; NrasG12D/+ ; PTEN+/+ (n=21) and Tyr::CreERt2/°; NrasG12D/G12D; PTEN+/+ (n=10) mice as well as ethanol-treated Tyr::CreERt2/°; NrasG12D/+; PTENf/+ (n=18), and Tyr::CreERt2/°; NrasG12D/G12D; PTENf/+ (n=17) mice. Median survival of +/G12D;+/PTEN mice is undefined and for G12D/G12D;+/PTEN is 10.5 months.
(B) Macroscopic images of NRASG12D/+ - ΔPTEN and NRASG12D/G12D - ΔPTEN tumours.
(C) Photomicrographs of hematoxilyn and eosin (H&E) stained skin from NRASG12D/+ - ΔPTEN and NRASG12D/G12D - ΔPTEN animals. Black arrows point to pigmented, dendritic melanocytes in the dermis. Scale bar = 50µm. (D) Photomicrographs of H&E stained tumours from NRASG12D/+ - ΔPTEN and NRASG12D/G12D - ΔPTEN animals. Black arrows point to mitotic cells. Scale bar = 300µm (left column), 50µm (right column). (E) Photomicrographs of immunohistochemistry (IHC) staining for Hmb45 (E), S100 (F), ppERK (G), pAKT (H) and Ki67 (I) from NRASG12D/+ - ΔPTEN and NRASG12D/G12D - ΔPTEN tumours. Scale bar = 50µm.

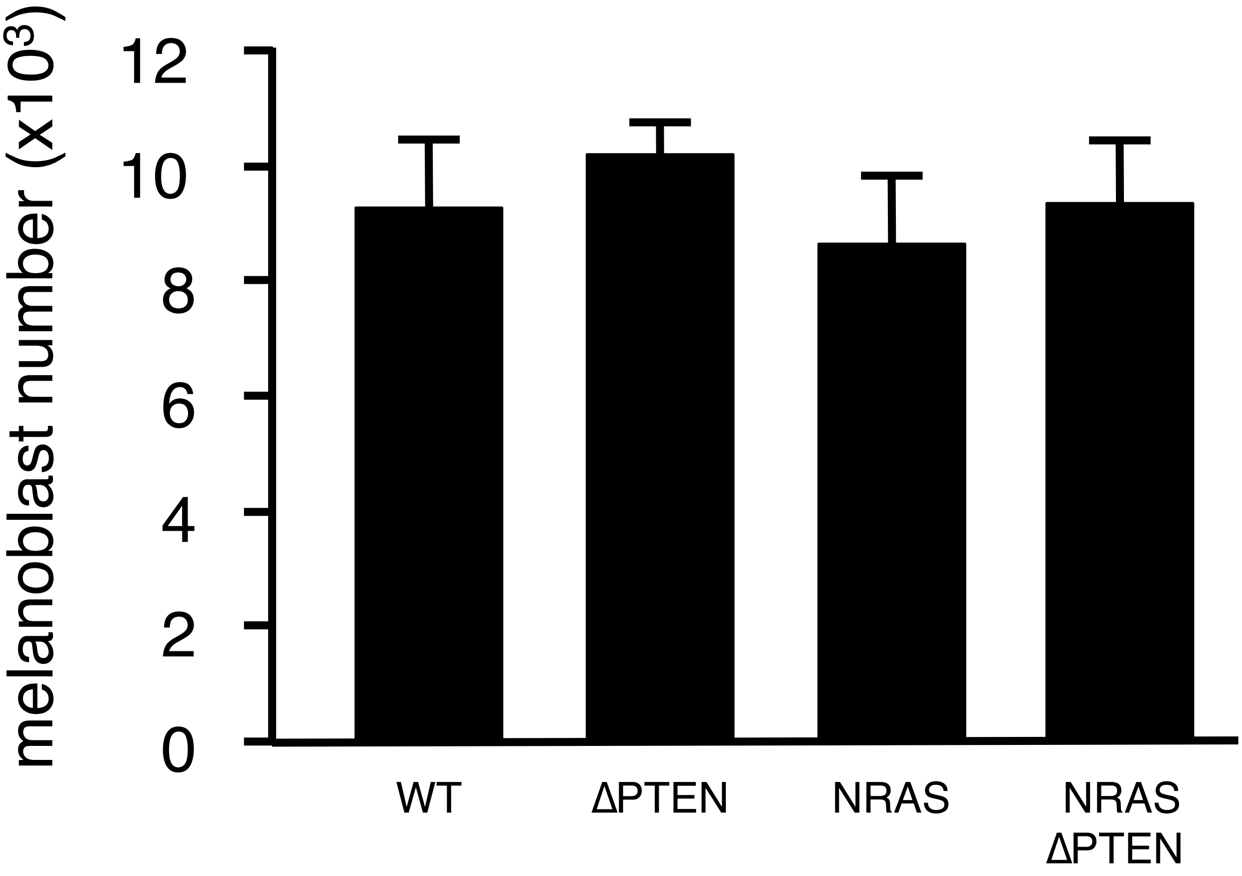

Supplementary Figure 7. Number of melanoblasts in embryos mutated for NRAS Q61K and lacking or not PTEN
During embryogenesis the lack of PTEN does not affect melanocyte precursor numbers. WT (Tyr::Cre/°; PTEN+/+; Dct::LacZ/°), ΔPTEN (Tyr::Cre/°; PTENf/f; Dct::LacZ/°), NRAS (Tyr::NRASQ61K; Tyr::Cre/°;PTEN+/+; Dct::LacZ/°) and NRAS-ΔPTEN (Tyr::NRASQ61K; Tyr::Cre/°; PTENf/f; Dct::LacZ/°) Dct-LacZ-positive cells were counted at E15.5 (n=5 for each genotype) at the trunk level of the embryos between the fore- and hind-limbs (somites 13 to 25) on the right side.

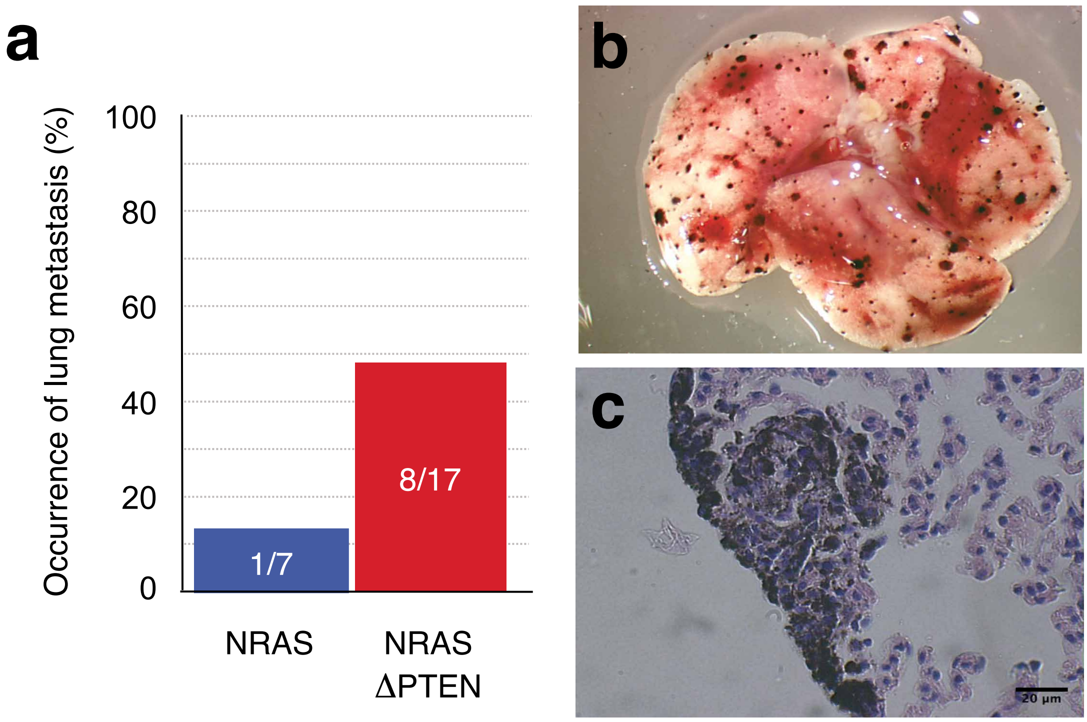

Supplementary Figure 8 . Lung melanoma metastasis
(A) Occurrence of lung metastasis in NRAS and NRAS-ΔPTEN mice. (B) Multiple lung melanoma metastases in NRAS-ΔPTEN mice. (C) Histological staining with haematoxylin and eosin showing pigmented cells, consistent with the diagnosis of melanoma lung metastasis. Scale bar = 20µm.

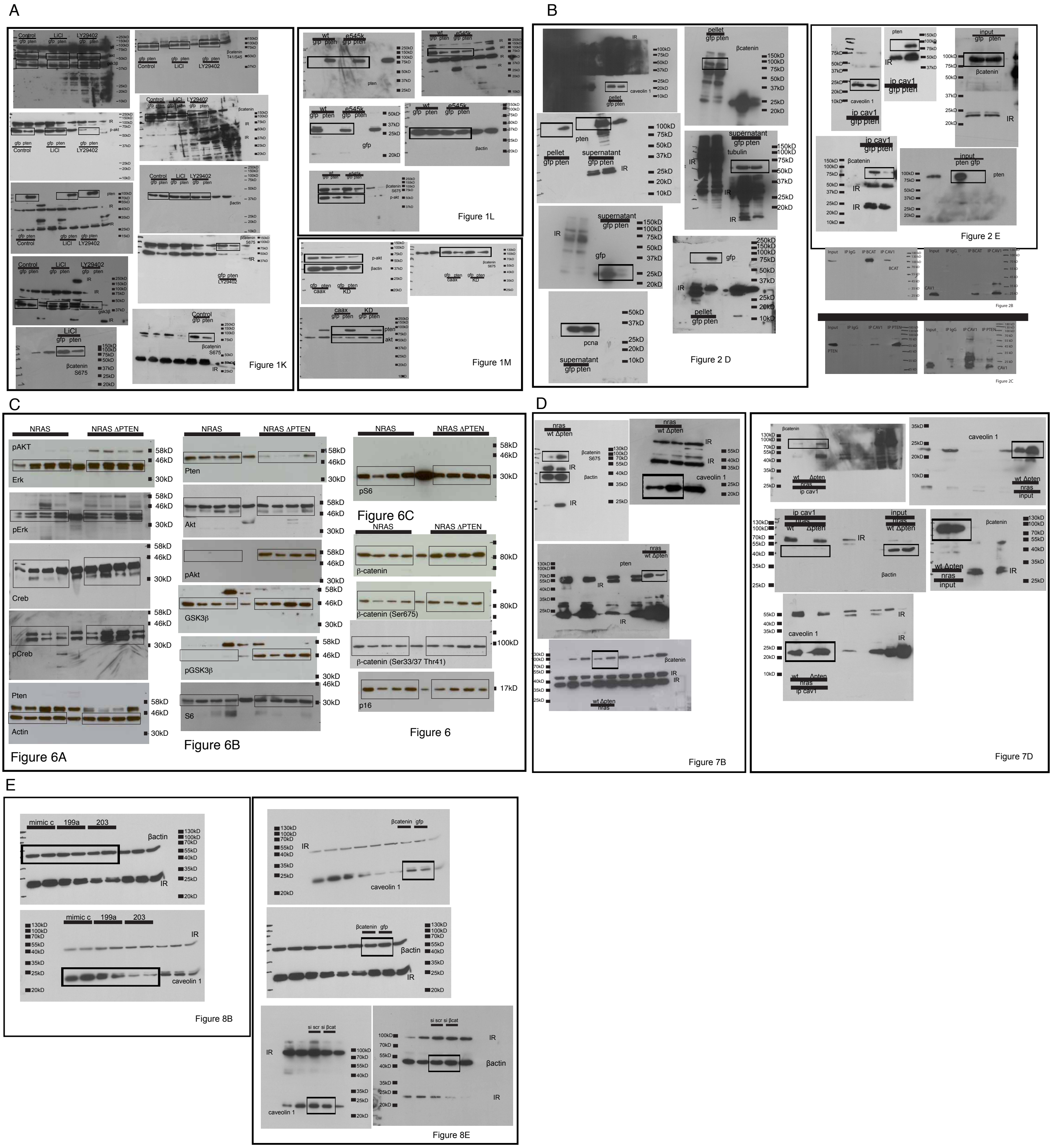

Supplementary Figure 9. Scans of the blots presented in the article by Conde-Perez et al.

(A) Scans of the blots presented in Figure 1K-M
(B) Scans of the blots presented in Figure 2B-E
(C) Scans of the blots presented in Figure 6A-C
(D) Scans of the blots presented in Figure 7B,D
(E) Scans of the blots presented in Figure 8B,E

Supplementary Table 1 Melanomas arising from different parts of the body

	NRAS	NRAS-ΔPTEN
Hairy skin	86.2% (25/29)	82.5% (52/63)
Pinnae	3.4% (1/29)	9.5% (6/63)
Tail or paw	6.9% (2/29)	3.2% (2/63)
Mucosa	3.4% (1/29)	4.8% (3/63)
